# Lysine demethylase 2 (KDM2B) regulates hippo pathway via MOB1 to promote pancreatic ductal adenocarcinoma (PDAC) progression

**DOI:** 10.1186/s13046-019-1489-0

**Published:** 2020-01-15

**Authors:** Ming Quan, Zhiqin Chen, Feng Jiao, Xiuying Xiao, Qing Xia, Jingde Chen, Qian Chao, Yandong Li, Yong Gao, Haiyan Yang, Liwei Wang, Jiujie Cui

**Affiliations:** 1 Department of Oncology and Tumor Institute, Shanghai East Hospital, Tongji University School of Medicine, Shanghai, China; 2 Department of Medical Oncology, Renji Hospital, School of Medicine, Shanghai Jiao Tong University, Shanghai, China; 3 State Key Laboratory of Oncogene and Related Genes, Shanghai Cancer Institute, Renji Hospital, School of Medicine, Shanghai Jiao Tong University, Shanghai, China

**Keywords:** Pancreatic Cancer, MOB1, KDM2B, Hippo signaling

## Abstract

**Background:**

Mps1 binding protein (MOB1) is one of the core components of the mammalian Hippo pathway and plays important roles in cancer development. However, its expression, function and regulation in pancreatic ductal adenocarcinoma (PDAC) have not been revealed yet.

**Methods:**

The expression of MOB1 and lysine demethylase 2B (KDM2B) in PDAC and adjacent normal pancreas tissues were measured. Also, the underlying mechanisms of altered MOB1 expression and its impact on PDAC biology were investigated.

**Results:**

We revealed for the first time that MOB1 was decreased expression in PDAC and was a statistically significant independent predictor of poor survival, and restored expression of MOB1 suppressed the proliferation, migration and invasion of PDAC cells. Further studies demonstrated that KDM2B directly bound to the promoter region of MOB1, and suppressed the promoter activity of MOB1 and transcriptionally inhibited the MOB1 expression. Furthermore, KDM2B regulated Hippo pathway and promoted PDAC proliferation, migration and invasion via MOB1.

**Conclusion:**

This study demonstrated the mechanism and roles of a novel KDM2B/MOB1/Hippo signaling in PDAC progression.

## Background

Despite decades of effort, the five-year survival rate of pancreatic ductal adenocarcinoma (PDAC) remains about 8%, and it has been estimated that PDAC would be the second leading cause of cancer-related death by 2030 [1, 2]. PDAC is the most common type of pancreatic malignancy (accounting for 95% of all cases), and about 85% of the patients have already spread locally or to distant organs at the time of diagnosis. Among patients undergoing a potentially curative resection, the outcome remains poor due to early recurrence and metastasis. To make things more difficult, effective systemic therapy is currently not available in PDAC. Thus, it is important to demonstrate the molecular mechanism and pathways promoting the development and progression of PDAC, and identify new targets for the treatment of PDAC.

Mps binding protein 1 (MOB1) is considered to be one of the core components of the mammalian Hippo pathway [3]. *MOB1A* and *MOB1B*, which have 95% sequence identity, play redundant biological roles and are both termed *MOB1* [4, 5]. MOB1 is an adaptor protein with no apparent functional domain and acts as co-activator of large tumor suppressor 1 and 2 (LATS1/2) kinases [5, 6]. In the presence of MOB1, LATS1/2 phosphorylate Yes Associated Protein (YAP) and/or its paralogue transcriptional co-activation with PDZ-binding motif (TAZ). Phosphorylated YAP and TAZ can bind to 14–3-3 protein which leads to the cytoplasmic retention of them, or be ubiquitinated and degraded in the cytoplasm [7–9]. Therefore, phosphorylated YAP and TAZ lose the activity in cell proliferation and anti-apoptosis [10, 11]. YAP and TAZ were reported to be the prognostic markers of PDAC and they promoted PDAC development and progression [12, 13]. Via regulating the protein levels and activity of YAP/TAZ, MOB1 acts as a tumor suppressor and loss of MOB1 promotes cell proliferation and induces cancers [4, 6, 14–16]. In PDAC, it has been reported that intrinsic programmed cell death protein 1(PD-1) bound to MOB1 and inhibited MOB1’s phosphorylation which increased the activation of YAP and promoted PDAC progression [17]. It has been reported that ubiquitin ligase praja2 ubiquitylated and degraded MOB1 and promoted glioblastoma growth [18]. In Hein AL et al’s study, they showed that PP2A inhibited the MOB1/LATS cascade, activated YAP and promoted PDAC progression [19]. However, the expression, roles and regulatory mechanism of MOB1 in PDAC have not been studied.

Epigenetic mechanisms, which are maintained by dynamic histone and DNA modifications by a group of chromatin-modifying enzymes, are central to regulate initiation and progression of cancers. These chromatin-modifying enzymes include histone acetyltransferases, histone deacetylases (HDACs), histone methyltransferases (HMTs), histone demethylases (HDMs) and DNA methytransferases (DNMTs) [20]. Altered activity of HDMs is emerging as a common defect [21]. Recently, studies have shown that lysine demethylase 2B (KDM2B) is an important regulator of cancer development and progression [22–24]. KDM2B, which is also known as Ndy1, FBXL10, and JHDM1B, can lead to demethylate of H3K36me2 and transcriptionally regulate the expression of genes [25]. In PDAC, Bardeesy N group’s study demonstrated that KDM2B promoted PDAC progression via polycomb-dependent and -independent manner [26]. Genes bound by KDM2B and Enhancer of zeste homolog 2(EZH2) are involved in developmental and pluripotency networks, whereas KDM2B-KDM5A and/or MYC co-bound genes mostly participate in metabolic processes [26]. However, the contribution of KDM2B to the development and progression of PDAC remains to be fully elucidated.

In the present study, we investigated the expression, roles and regulatory mechanism of MOB1 in PDAC. We showed that restored expression of MOB1 inhibited PDAC cell proliferation, migration and invasion. Further mechanism study revealed that KDM2B directly bound to the promoter region of *MOB1* gene, led to the methylation of H3K27 and repressed the expression of MOB1, and promoted PDAC progression via Hippo signaling.

## Methods

### Human tissue specimens and immunohistochemical analysis

The expression of MOB1, KDM2B, and YAP were analyzed using tissue microarrays (TMA) from Shanghai Outdo Biotech Company (China). The TMA contains 100 primary PDAC tissues, 80 adjacent normal pancreas tissues. All the samples were from patients who received surgeries. And all the patients did not receive prior chemotherapy or radiotherapy. Clinical and demographic information, including age, gender, tumor localization, clinical staging, differentiation grade, invasive to the vessel, invasive to the nerve, tumor size and survival from the time of diagnosis were available with patients consent. Immunohistochemical analysis was conducted with anti-MOB1 (SAB4301095, Sigma-Aldrich, St. Louis, MO, USA, diluted 1:200), anti-KDM2B (SAB2702002, Sigma, USA, diluted 1:300) and anti-YAP (#12395, Cell Signaling Technology, diluted 1:300) antibodies. The evaluation of immunohistochemistry was performed as reported [27]. Berifly, MOB1, KDM2B and YAP immunostaining signals were evaluated by two pathologists blind to the clinical information. The percentage of MOB1-, KDM2B- or YAP-positive cells was scored into the following four categories: 1 (< 25%); 2 (25 to 50%); 3 (50 to 75%); and 4 (> 75%). The staining intensity of positive cells was scored as 0 (absent); 1 (weak infiltration); 2 (moderate infiltration), and 3 (strong infiltration). The final score was the product of the intensity and the percentage. For statistical analyses, these categories were further dichotomized into MOB1/KDM2B/YAP-Low expression (0–4) or -High expression (6–12).

### Cell lines and reagents

The human PDAC cell lines PANC-1, MiaPaCa-2, AsPC-1, BxPC-3, SW1990 and immortalized human pancreas duct epithelial cell line hTERT-HPNE cells were purchased from the American Type Culture Collection (ATCC). FG were described previously [28]. All of these cancer cell lines were maintained in plastic flasks as adherent monolayers in Eagle’s minimal essential medium supplemented with 10% fetal bovine serum, sodium pyruvate, nonessential amino acids, L-glutamine, and a vitamin solution (Flow Laboratories). hTERT-HPNE cells were cultured in a mixture of Dulbecco’s Modified Eagle’s Medium without glucose (Sigmaich-Aldrich, Cat. No. D-5030) and Medium M3 Base (Incell Corp, Cat. No. M300F-500) (3:1 ratio) with 2 mML-glutamine adjusted to 1.5 g/L sodium bicarbonate and supplemented with 5% FBS, 10 ng/ml human recombinant EGF, 5.5 mM D-glucose (1 g/L) and 750 ng/mL puromycin. The cell lines were obtained directly from ATCC that performs cell line characterizations or authentication by the short tandem repeat profiling and passaged in our laboratory for fewer than 6 months after receipt.

The following drugs were used with an indicated concentration in the experiments: MST1/2 activator okadaic acid (OA) and de-methylating agent 5-aza-2′-deoxycytidine (5-aza) were purchased from Sigma [3, 29].

### Plasmids and short hairpin RNAs (shRNA)

Both the plasmids pcDNA3.0/HA-tagged MOB1 (pMOB1) (Plasmid #32835) and 8xGTIIC-luciferase (Plasmid #34615) which is the YAP/TAZ luciferase reporter were obtained from Addgene [7, 30]. A 408 bp fragment containing *MOB1* transcrptional start sites (TSSs) which is 5′ sequences from − 455 to − 47 relative to the starting codon was subcloned into the pGL3-basic vector (Promega). The sequences of the primers were as follows: 5′-TCG GGG TAC CCC GCG CTT ATA GAG GTC CCT GCA TAA C-3′ (forward) and 5′-TCC CAA GCT TGG GTC AGG ATT CGG AGC TGG CTA G-3′ (reverse). The following are the siRNA sequences were synthesized targeting human *MOB1*: sequence 1: 5′-AUG AAU GGG UUG CAG UUA A-3′; sequence 2: 5′-GCA GAU GGA ACG AAC AUA A-3′. ShRNA constructs with the target sequences of *KDM2B* sequence 1:5′-GAT GAG CAT GTC CCA GTT T-3′; sequence 2: 5′-TGA GCG TGA AAG GTT GTT T-3′were reported previously [18, 31, 32]. Each amplified DNA fragment was verified by sequencing the inserts and flanking regions of the plasmids.

### Quantitative real-time RT-PCR

Quantitative real-time RT-PCR analysis of the expression of the MOB1 was performed using total RNA and the SYBR Green reagent with an ABI Prism 7000HT sequence detection system (Applied Biosystems) [33]. The primers used in the reaction for MOB1 and β-actin were designed and synthesized by Tiangen (Tiangen, Beijing, China). The sequences of the PCR primers were as follows: MOB1A, 5′-ATT TCG GGT CTG CGA GGT G-3′ (forward) and 5′-GGA TCA GGA TTC GGA GCT GG-3′ (reverse); MOB1B: 5′-CCT CCC TGT CTC CTG TTC CAT-3′ (forward) and 5′-GCT GAG AAC TCA ACA AGA AGC TC-3′ (reverse); β-actin, 5′-AGC CGG GCT CTT GCC AAT-3′ (forward) and 5′-AGT TAG CGC CCA AAG GAC CA-3′ (reverse). The experiments were performed in triplicate and repeated twice.

### Gene transfection

For transient transfection, cells were transfected with plasmids or shRNA for 48 h before functional assays using Lipofectamine LTX and Lipofectamine 2000 CD (Invitrogen), respectively. PDAC Cells transfected with control plasmids or shRNA using Lipofectamine LTX and Lipofectamine 2000 CD were defined as controls. PDAC cells treated with the transfection reagents alone were included as mock controls.

### Preparation of cytoplasmic and nuclear protein fractions and western blot analysis

Total cell lysates were extracted. For the preparation of cytoplasmic and nuclear protein fractions, the PDAC cells were scraped from culture plates with cold phosphate-buffered saline (PBS) and resuspended in hypotonic lysis buffer (10 mM HEPES, pH 7.9, 10 mM KCl, 1.5 mM MgCl2, 0.5 mM dithiothreitol). The cells were then Dounce-homogenized and centrifuged at 1000×g for 5 min at 4 °C. The supernatant (cytoplasmic fraction) was stored at − 80 °C until use. The nuclei in the pellet were isolated via centrifugation and resuspended in nuclear extraction buffer (20 mM HEPES, pH 7.9, 420 mM NaCl, 1.2 mM MgCl2, 0.2 mM ethylenediaminetetraacetic acid, 25% glycerol) for 30 min at 4 °C. After centrifugation at 15,000×g for 30 min at 4 °C, the supernatant (nuclear fraction) was stored at − 80 °C.

Standard Western blotting was carried out using primary anti-MOB1 (SAB4301095, Sigma), anti-KDM2B (SAB2702002, Sigma), anti-YAP (#12395, Cell Signaling Technology), pYAP (#13008, Ser127, Cell Signaling Technology), TAZ (#4883, Cell Signaling Technology), pTAZ (#59971, Ser89, Cell Signaling Technology), MST1(#3682, Cell Signaling Technology), pMST1(#49332, Thr183, Cell Signaling Technology), SAV1 (#13301, Cell Signaling Technology), LATS1 (#3477, Cell Signaling Technology), pLATS1(#9157, ser901, #8654 Thr 1079, Cell Signaling Technology), anti-H3K27me3 (#9733, Cell Signaling Technology). Equal protein-sample loading was monitored using an anti-β-actin antibody for total cell protein lysates (rabbit; Santa Cruz Biotechnology), an anti-α-tublin antibody (for total cell protein lysates and cytoplasmic fractions; Oncogene, Rockville, MD, US), and an anti-Histone H1 antibody (for nuclear fractions; Santa Cruz). Secondary antibodies were anti-mouse IgG, anti-rabbit IgG or anti-goat IgG (Santa Cruz Biotechnology). The bands were quantified using the Quantity One analysis software program (version 4.6; Bio-Rad). The experiments were repeated twice.

### Chromatin immunoprecipitation assay

Tumor cells (2 × 10^6^) were prepared for chromatin immunoprecipitation (ChIP) assay with the ChIP assay kit (Millipore Technology, Billerica, MA) according to the manufacturer’s protocol. The resulting precipitated DNA samples were analyzed using quantitative real-time PCR to amplify a 408-bp region of the MOB1 promoter with the primers 5′-CGC TTA TAG AGG TCC CTG CAT AAC-3′ (forward) and 5′-TCA GGA TTC GGA GCT GGC TAG-3′ (reverse); a 80-bp region of the YAP promoter with the primers 5′-TCG CCG CTT CTC CAC CT-3′(forward) and 5′- GAC GCG CAC CCC CTG AC-3′ (reverse); a 101-bp region of the TAZ promoter with the primers 5′- AGA AAC CTC AGA GTG ACT AAA AT − 3′(forward) and 5′- TAA GAG TCA GAT GAG CAG AGA TG − 3′ (reverse). The antibodies used in the ChIP assay were anti-KDM2B (#09–864, Millipore), anti-H3K27me3 (#9733, Cell Signaling Technology), anti-H3K36me2 (#2901, Cell Signaling Technology), anti-H3K4me3 (#9751, Cell Signaling Technology). The experiments were performed in triplicate and repeated twice.

### Colony-formation assay

Two hundred cells from each group as indicated were plated in 24-well plates and allowed to grow for 14 days in culture medium; the medium was changed twice a week. Cells were then fixed with 4% paraformaldehyde and stained with 0.1% crystal violet solution for 10 min. Colonies (>20 cells) were counted using an inverted microscope at 40× magnification. The results were calculated as the percentage of proper control. The experiments were performed in triplicate and repeated three times.

### Measurement of cell migration and invasion

Cell migration and invasion assays were performed with the Cell migration (ECM509) and Invasion (ECM554) Assay Kits which were obtained from ECMatrix. Briefly, 24 h after different treatments in different groups, the cells were harvested and suspended in DMEM at a concentration of 8 × 10^4^ /ml. Cells prepared in 500 μl of DMEM were loaded in the upper wells and a medium containing 20% FBS was placed in the lower wells as a chemoattractant stimulus. Migrated cells on the bottom surface of the filter were fixed, stained with H&E, and counted under a microscope in three randomly selected fields at a magnification of 200×. The experiments were performed in triplicate and repeated three times.

### Co-Immunoprecipitation

Proteins were extracted from cell lysates with lysis buffer. Protein A/G beads (Thermo Fisher Scientific, Waltham, MA, USA) and anti-HA (#3956, Cell Signaling Technology) were added to protein lysates, then incubated overnight. Proteins from the beads were subjected to western blot.

### Mouse model of pancreatic tumor growth

Male Balb/c nude mice were purchased from Sippr-BK Laboratory Animal Corporation, Shanghai, China. PDAC cells (1 × 10^6^) in 0.1 mL of Hank’s balanced salt solution were injected subcutaneously of nude mice. The tumor size was measured every week. The tumor-bearing mice were sacrificed when they on day 35 after inoculation and the tumors were removed and weighed. All animal experiments were performed according to the protocols approved by the Medical Experimental Animal Care Commission of Tongji University.

### Cell immunofluorescence

Transfected PANC-1 cells were then further cultured on Falcon chamber slides (BD Biosciences) at up to 30–40% confluence before being fixed with 4% paraformaldehyde and permeabilized with 0.3% Triton X-100. The cells were then immersed three times in phosphate-buffered saline, incubated with indicated anti-YAP (#12395, Cell Signaling Technology) and anti-TAZ (#4883, Cell Signaling Technology) primary antibody overnight at 4 °C and corresponding Alexa Fluor-conjugated secondary antibodies (Invitrogen) for 1 h at room temperature, and mounted using a mounting medium containing 4′,6- diamidino-2-phenylindole. Microscopic images of cells were obtained using an Axio Observer A microscope (Zeiss). The experiments were performed in triplicate and repeated twice.

### Statistical analysis

The significance of the patient specimen data was determined using the Pearson correlation coefficient. The two-tailed χ^2^ test or Fisher exact test was used to determine the significance of the differences among the covariates. Overall survival (OS) was defined as the interval from the date of diagnosis until death from any cause. The OS was estimated using the Kaplan-Meier method and compared using the log-rank test. Significant variables were further analyzed by multivariate analysis to test for independent prognosis. Bivariate correlations between variable factors were calculated by Spearman rank correlation coefficients. All the in vitro experiments were repeated at least once, and one representative experiment of two or three with similar results were presented; the data were shown as mean ± SD or as indicated properly, and the significance of the data was determined using the Student *t-test* (two-tailed) or one-way analysis of variance. In all tests, *P* values less than 0.05 were considered statistically significant. The SPSS software program (version 13.0; IBM Corporation) was used for statistical analysis.

## Results

### Suppressed expression of MOB1 directly related with pathologic features in PDAC

**Fig. 1:**
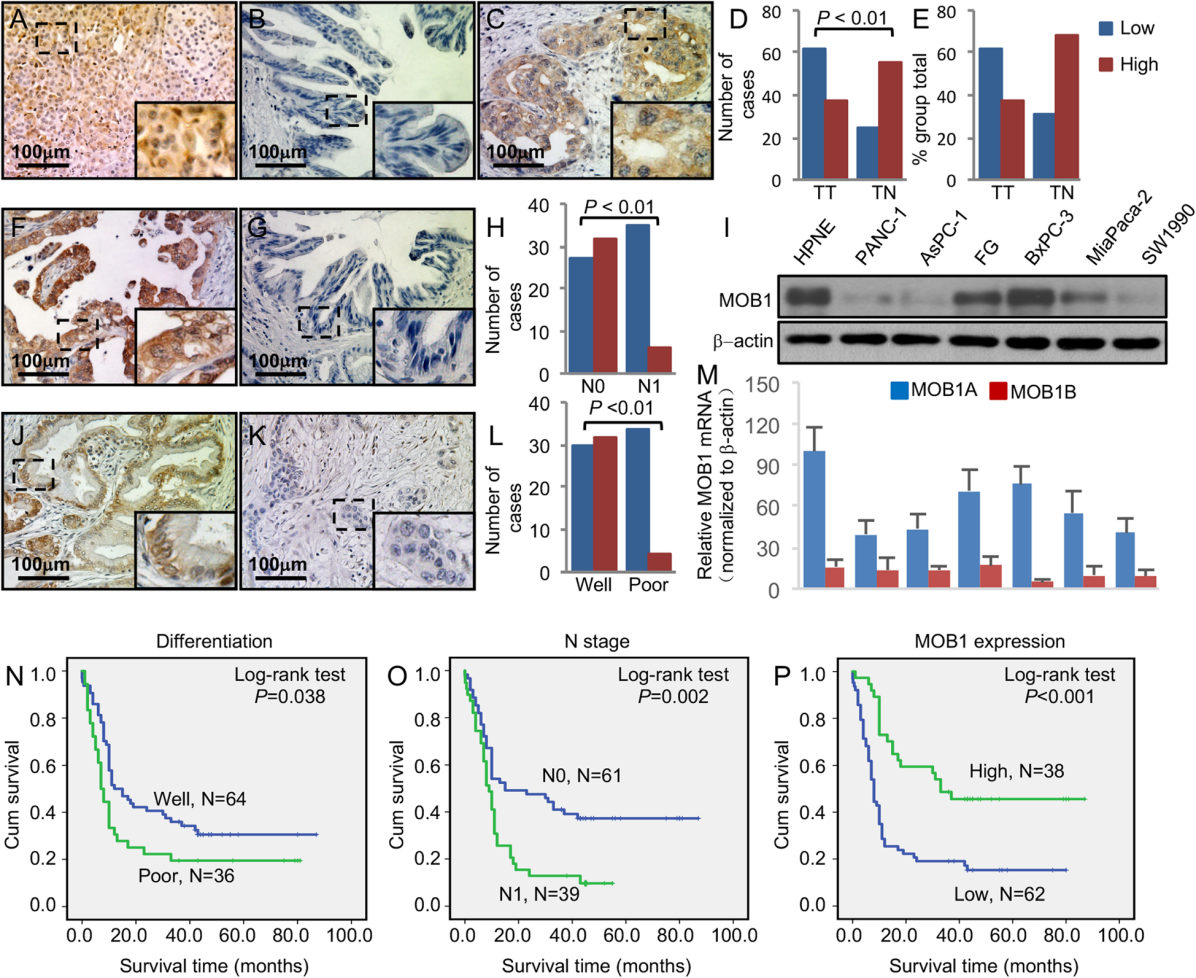
Expression of MOB1 in and its association with clinicopathologic features of PDAC. TMA PDAC specimens were immunostained with a specific anti-MOB1 antibody. **a**, representative images of MOB1 expression in adjacent normal pancreatic tissue specimens. **b**, representative image of low MOB1 expression in PDAC specimens. **c**, representative image of high MOB1 expression in PDAC specimens. **d** and **e**, the expression of MOB1 was significantly lower in tumors (TT) than in adjacent normal tissue (TN). **f** and **g**, representative images of high MOB1 expression in PDAC specimens without lymph nodes metastasis and low MOB1 expression with lymph nodes metastasis. **h**, the expression of MOB1 was negatively associated with lymph nodes metastasis. **i** and **j**, representative images of high MOB1 expression in PDAC specimens of well differentiation and low MOB1 expression of poor differentiation. **k**, the expression of MOB1 was negative associated with PDAC poorer differentiation. **l**, the protein levels of MOB1 in PDAC cell lines and normal pancreas cells (HPNE). **m**, the mRNA levels of MOB1A and MOB1B in PDAC cell lines and normal pancreas cells. **n**, **o** and **p**, the tumor differentiation, N stages and MOB1 expression were associated with the OS of PDAC patients

To determine the role of MOB1 expression in PDAC pathogenesis, we first investigated the expression level of MOB1 in PDAC tissue array via IHC (The clinicopathologic characteristics of the tissue array were described in Additional file 1: Table S1). We observed MOB1-positive staining mainly in the cytoplasm of adjacent normal pancreatic tissues and some cancer tissues, and it showed a decreasingly positive staining activity of MOB1 among cancers (Fig. 1a, b, c, d, e). The decreased expression of MOB1 was positively associated with tumor lymph nodes metastasis (Fig. 1f, g, h, and Additional file 1: Table S1), tumor poor differentiation (Fig. 1i, j, k and Additional file 1: Table S1) and clinical stages (Additional file 1: Table S1). Kaplan-Meier analysis and log-rank test were used to investigate the prognostic value of MOB1 expression and classic clinicopathologic characteristics on patient survival. In univariate analysis, MOB1 was closely associated with overall survival (OS) of PDAC patients (*P* < 0.001; Fig. 1p and Additional file 4: Table S4). Univariate analysis also indicated that tumor differentiation (*P* = 0.038; Fig. 1n and Additional file 4: Table S4), lymph nodes metastasis (*P* = 0.002; Fig. 1o and Additional file 4: Table S4), and clinical stages (*P* = 0.001; Additional file 4: Table S4) were correlated with patient OS. Multivariate analysis showed that MOB1, lymph nodes metastasis and clinical stages were independent prognostic factors for PDAC patients (Additional file 4: Table S4). We then further tested the expression of MOB1 in PDAC cell lines via western bolt. The levels of MOB1 were obviously lower in most cancer cell lines (Fig. 1l). MOB1A and MOB1B both termed MOB1 and with over 95% sequence identity. It difficult to identify MOB1A and MOB1B with antibodies, thus we tested the mRNA levels of MOB1A and MOB1B with q-PCR in PDAC cells lines. We found that the levels of MOB1A was lower in PDAC cell lines than HPNE, and MOB1B was at a quite low levels in both PDAC and normal cell lines than MOB1A, which indicated that it was MOB1A playing a dominate role as MOB1 in PDAC (Fig. 1m). Thus, we used MOB1A to stand for MOB1 in the further study.

These findings revealed that lost expression of MOB1 played critical roles in PDAC and MOB1 might be a valuable biomarker for this disease.

### Effect of MOB1 on PDAC proliferation, migration and invasion

**Fig. 2:**
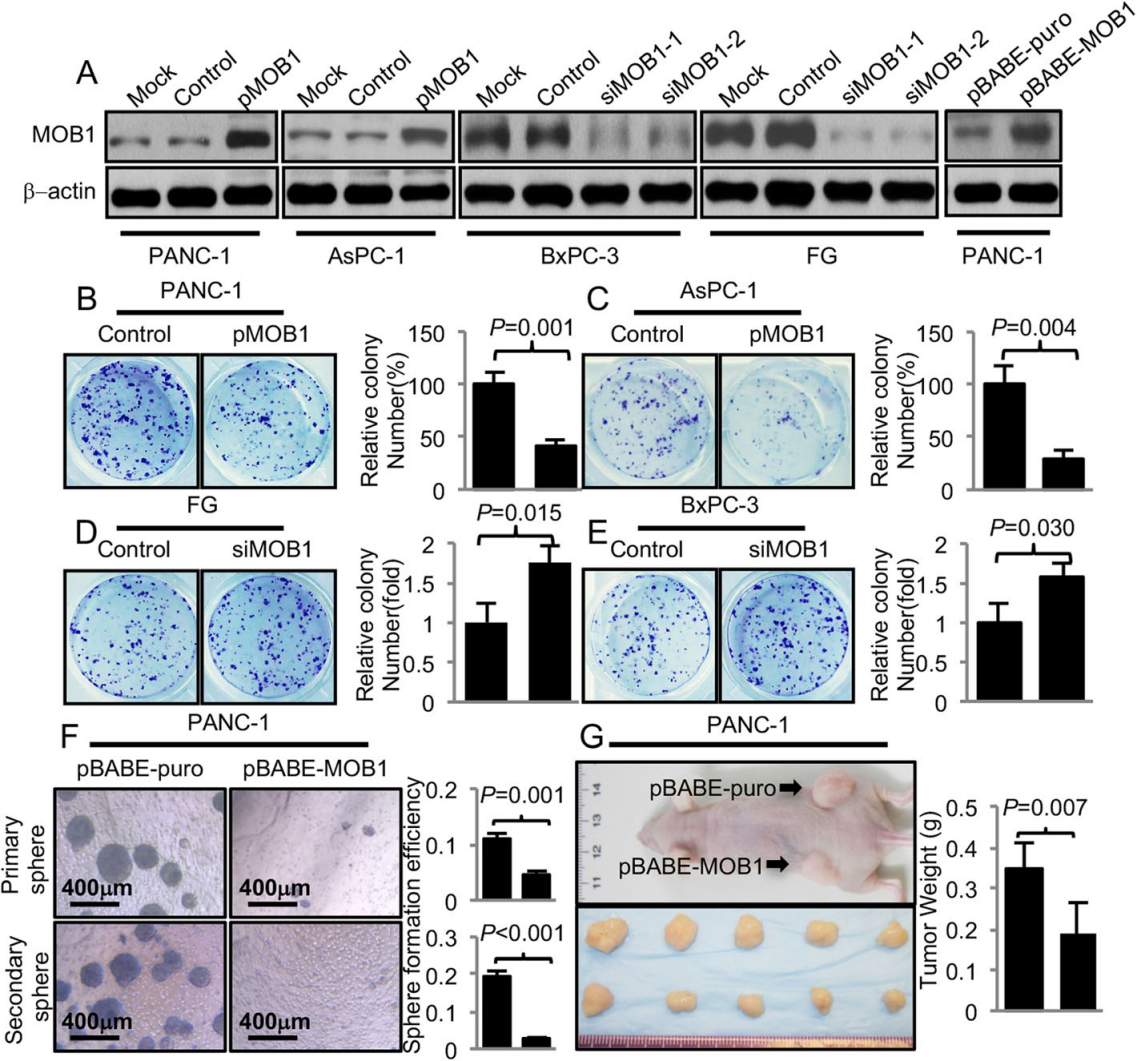
Effect of altered MOB1 expression on PDAC biology. **a**, verification of the efficiency of MOB1 overexpression vectors and siRNAs in PDAC cell lines. PANC-1 and AsPC-1 cells were transfected with pMOB1 or control vectors. BxPC-3 and FG cells were transfected with siMOB1–1 and siMOB1–2 or control siRNAs. PANC-1 cells were infected with retroviruses containing MOB1and empty retroviral expression vectors used as controls. Then infected PANC-1 cells were selected with puromycin. The protein levels of MOB1 were tested via western blot. Relative expression levels normalized to β-actin were shown in fold below the bands. PANC-1 and AsPC-1 cells were transfected with pMOB1 or control vectors, and FG and BxPC-3 cells were transfected with siMOB1–1(siMOB1) or control siRNAs for 24 h. **b**, **c**, **d** and **e**, colony formation assay was performed with PANC-1, AsPC-1, FG and BxPC-3 cells with different treatment in 24-well plates and numbers of colonies were counted 14 days after. **f**, spheroid colony formation assay was performed with PANC-1 cells, and restored expression of MOB1 decreased the number and size of the first and second generations of spheroids. PANC-1 cells with restored expression of MOB1 were injected subcutaneously (1 × 10^6^ per mouse, *n* = 5) into the right and left scapular region of the nude mice. The tumor-bearing mice were sacrificed when they became moribund or on day 35. **g**, shown was representative photo of mouse and gross tumors in the mice and the weight of the tumors was measured

To assess the impact of MOB1 expression on PDAC cancer cell biology, we transfected PANC-1 and AsPC-1 cells, which had very low or intermediate levels of endogenous MOB1, with pcDNA3.0/HA-tagged MOB1 (PANC-1/AsPC-1-pMOB1), and pcDNA3.0 was used as control (Fig. 2a). Furthermore, we infected PANC-1 cells with retroviruses containing MOB1 (PANC-1-pBABE-MOB1) and empty retroviral expression vectors used as controls (PANC-1-pBABEpuro). After selection of the infected cells with puromycin, we found that the levels of MOB1 were significantly elevated in the pooled drug-resistant cells (Fig. 2a). Conversely, we knocked down MOB1 using specific siRNAs against MOB1 in BxPC-3 and FG cells, and found that both siMOB1–1 and siMOB1–2 could effectively knock down MOB1 (Fig. 2a and Additional file 5: Figure S1) and we selected siMOB1–1 as siMOB1 for further study (Fig. 2a).

We then tested the effect of MOB1 on PDAC cell proliferation in vitro. As shown in Fig. 2b, and c, restored expression of MOB1 significantly inhibited the proliferation of PANC-1 and AsPC-1 cells via colony formation assay, and knockdown of MOB1 increased the number of colonies (Fig. 2d and e). The role of MOB1 in PDAC cell spheroid formation was tested. Re-expression of MOB1 significantly decreased the number and size of the first and second generations of spheroids in PANC-1 cells (Fig. 2f). We then further determined the effect of re-expressed MOB1 on PDAC growth in vivo. We found that restored expression of MOB1 markedly suppressed tumor growth (Fig. 2g). These data indicated that MOB1 inhibited PDAC growth. Furthermore, we investigated the effect of MOB1 on PDAC migration and invasion. Similarly, restored expression of MOB1 suppressed the migration and invasion of PANC-1 and AsPC-1 cells, whereas knockdown of MOB1 increased the migration and invasion of FG and BxPC-3 cells (*, *P* < 0.05) (Additional file 6: Figure S2A and S2B).

Collectively, these data clearly demonstrated that MOB1 suppressed the proliferation, growth, migration and invasion of PDAC, and supported that MOB1 might function as a tumor suppressor in PDAC.

### MOB1 regulated hippo pathway in PDAC cells

**Fig. 3:**
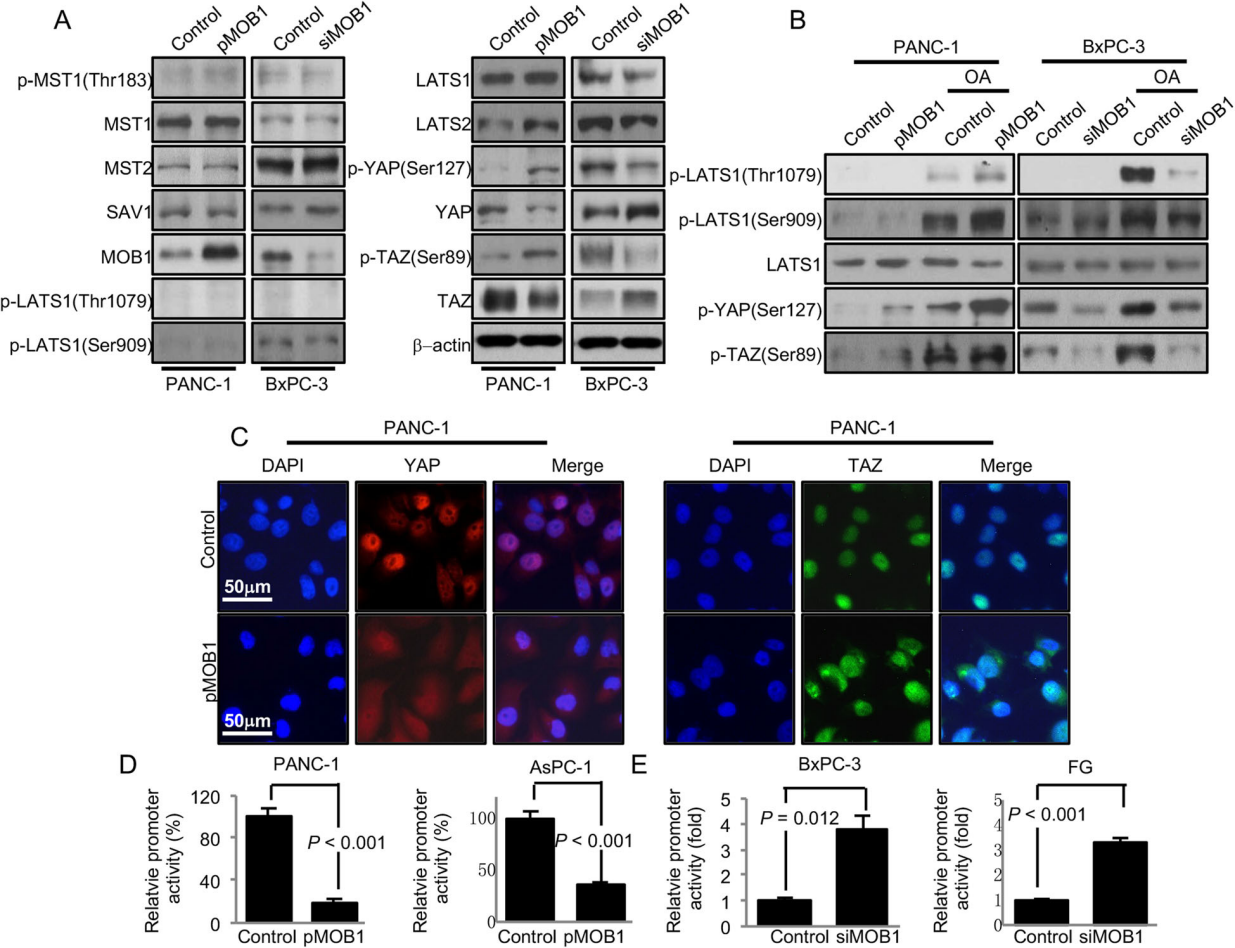
MOB1 regulated Hippo pathway in PDAC cells. **a**, PANC-1 cells were transfected with pMOB1 or control vectors, and BxPC-3 cells were transfected with siMOB1–1(siMOB1) or control siRNAs. The Hippo pathway proteins were analyzed with western blot. **b**, pLATS1, pYAP and pTAZ proteins were tested in MOB1 restored and knockdown cells that were untreated or treated with okadaic acid (OA, 10 μM) for 30 min. Total LATS1 protein levels in each sample were adjusted to equality before electrophoresis. **c**, PANC-1 cells were transfected with pMOB1 or control vectors for 24 h. Cell immunofluorescence was used to detect YAP (red) and TAZ (green) in PANC-1 at low cell density (nuclei were stained with DAPI (blue)). **d** and **e**, the 8xGTIIC-Luciferase reporter which is the YAP/TAZ-responsive synthetic promoter driving luciferase reporter was transfected into PANC-1, AsPC-1, BxPC-3 and FG cells in triplicate with MOB1 restored expression or siMOB1 or control vectors for 24 h. The promoter activity was examined by a dual luciferase assay kit

MOB1 is the core component of Hippo pathway, which plays critical role in PDAC development and progression [34, 35]. To further identify the molecular mechanisms underlying the tumor-suppressive function of MOB1 in PDAC, we focused on the impact of MOB1 on Hippo signaling components. Firstly, we verified whether MOB1 also bound to MST1/2 and LATS1/2 in PDAC cells. As shown in Additional file 7: Figure S3, MOB1 bound to MST1/2 and LATS1/2 in PANC-1 cells. We then tested the effect of MOB1 on the components of Hippo pathway. As expected, restored expression of MOB1 increased the phosphorylation of YAP (Ser127), TAZ (Ser89), LATS1 and LATS2, and reduced total YAP and TAZ proteins levels. Whereas knockdown of MOB1 decreased the levels of phospho-YAP (Ser127), phospho-TAZ (Ser89), LATS1 and LATS2, and increased total YAP and TAZ protein levels. No differences were found in the levels of phospho-MST1 (Thr183), MST1, MST2, SAV1, phospho-LATS1 (Thr1079) or phospho-LATS1 (Ser909) (Fig. 3a and Additional file 8: Figure S4A and S4B). We then stimulated cells with okadaic acid (OA), which activated MST1/2, and performed western blot after adjusting lysates to equality of LATS1 protein [29]. We found that OA-stimulated groups had vigorous LATS1 phosphorylation. And in OA-stimulated groups, restored expression of MOB1 further increased phospho-LATS1 (Thr1079), phospho-LATS1 (Ser909), phospho-YAP (Ser127) and phospho-TAZ (Ser89), but knockdown of MOB1 decreased the level of phospho-LATS1 (Thr1079), phospho-LATS1 (Ser909), phospho-YAP (Ser127) and phospho-TAZ (Ser89) (Fig. 3b and Additional file 8: Figure S4C and S4D). These data revealed that restored expression of MOB1 activated Hippo signaling in PDAC. It has been reported that YAP translocated into the nucleus at low cell density in normal and cancer cells [7, 36]. In our study, restored expression of MOB1 at low cell density also led to cytoplasmic localization of YAP and TAZ (Fig. 3c). We then analyzed the expression of YAP in the serial PDAC tissue array of MOB1 using IHC. We observed that in low MOB1 expression tissues, YAP was highly expressed and mainly positive staining in the nucleus of cancer tissues. But in high MOB1 expression tissues, the expression of YAP was low and mainly localized in the cytoplasm (Additional file 9: Figure S5A). Negative correlation of MOB1 and YAP with the same cohort was found in PDAC tissues (Additional file 9: Figure S5B, *r* = − 0.568, *P* < 0.001). Furthermore, increased expression of YAP was positively associated with poor tumor differentiation (Additional file 9: Figure S5C and S5D, and Additional file 2: Table S2, *P* < 0.001). Kaplan-Meier analysis and log-rank test were used to investigate the prognostic value of YAP on PDAC patient survival. In univariate analysis, the expression of YAP was negatively associated with the OS of PDAC patients (*P* < 0.001; Additional file 9: Figure S5F and Additional file 4: Table S4). Multivariate analysis showed that YAP was also an independent prognostic factor for PDAC patients (*P* = 0.001; Additional file 4: Table S4).

We further tested the effect of MOB1 on Hippo signaling pathway transcription. The results of luciferase assays showed that re-expression of MOB1 decreased the Hippo signaling transcriptional activity, whereas knockdown of MOB1 increased the transcriptional activity of the Hippo pathway (Fig. 3d and e). These data demonstrated that MOB1 inhibited the nuclear translocation and activity of YAP and TAZ, and further inhibited PDAC development and progression.

### KDM2B binding to the promoter region and transcriptionally suppressed the expression of MOB1

**Fig. 4:**
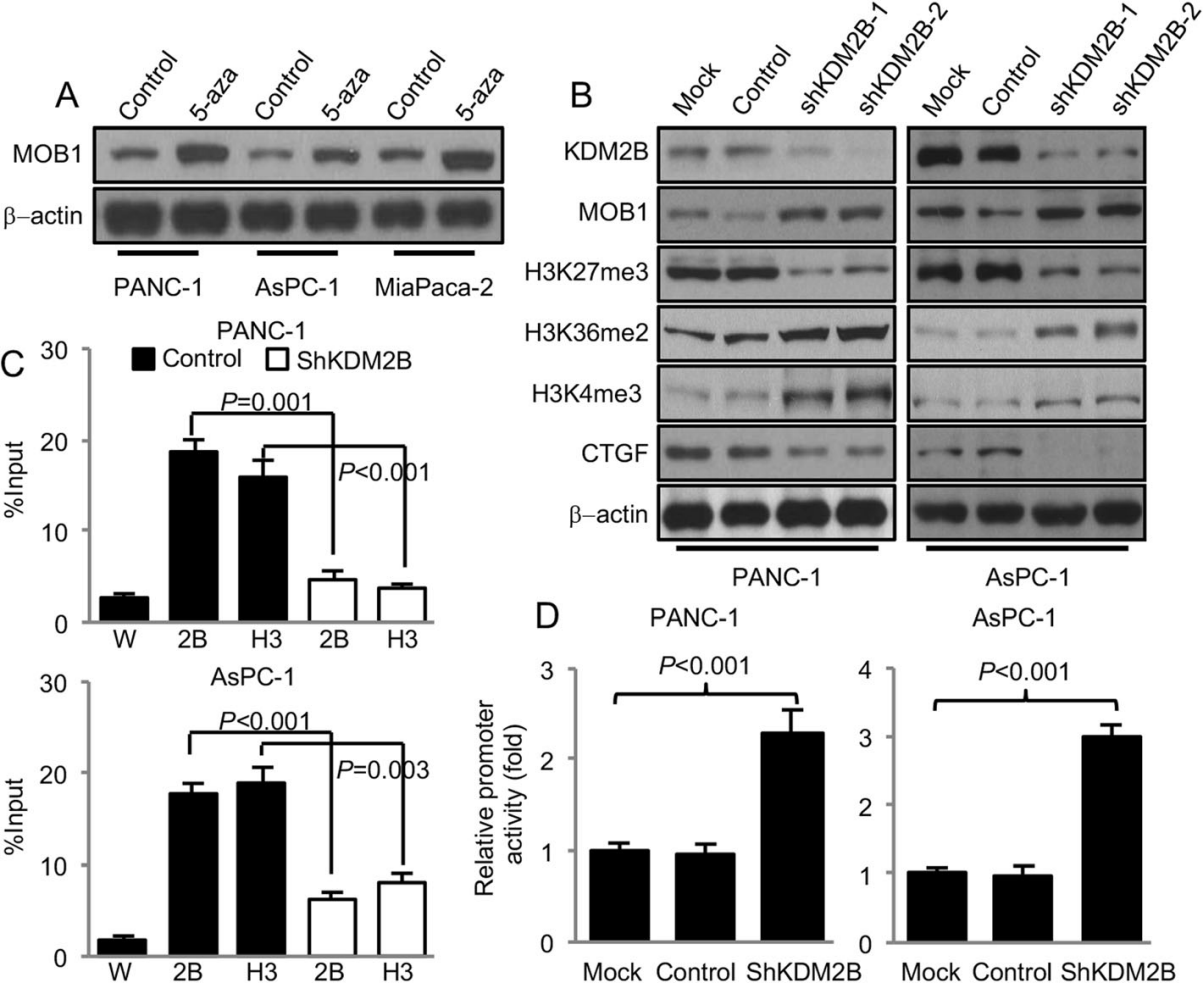
KDM2B transcriptionally suppressed the expression of MOB1. **a**, PANC-1, AsPC-1 and MiaPaca-2 cells were treated with de-methylating agent 5-aza at the concentration of 5 μM for 72 h. Protein levels of MOB1 were analyzed by western blot (β-actin as internal control). **b**, KDM2B was knocked down in PANC-1 and AsPC-1 cells using shRNAs, and the expression of MOB1, H3K27me3, H3K36me2 and H3K4me3 and CTGF were tested using western blot (β-actin as internal control). **c**, PANC-1 and AsPC-1 cells were transfected with shKDM2B or control vectors. Chromatins were isolated and the binding of KDM2B and H3K27me3 to the *MOB1* promoter was determined using ChIP assay as described in Materials and Methods (W: water; 2B: KDM2B; H3: H3K27me3). **d**, *MOB1* promoter reporter was constructed. PANC-1 and AsPC-1 cells were co-transfected with 0.2 μg of the *MOB1* promoter luciferase constructs pLuc-MOB1 and shKDM2B or control vector. Promoter activity was examined using a dual luciferase assay kit

We further studied the regulatory mechanisms of MOB1 in PDAC. It has been reported that CpG mehtylation was detected in *MOB1* promoter [37]. Thus, we incubated PANC-1, AsPC-1 and MiaPaca-2 cell lines with the demethylating agent 5-azacytidine (5-aza) for 72 h and western blot was used to test the protein levels of MOB1. The result revealed that 5-aza increased MOB1 (Fig. 4a and Additional file 10: Figure S6A). These data indicated that DNA hypermethylation in the promoter region of *MOB1* might also inhibit the expression of MOB1 in PDAC. In Bardeesy N group’s study, KDM2B cooperated with Kras and drove PDAC development [26]. In their study, knockdown of KDM2B decreased the levels H3K27me3, and increased the levels of H3K36me2 and H3K4me3 [26]. The study also showed that KDM2B bound to the transcriptional start sites (TSSs), decreased H3K27me3, methylated DNA of promoter region, and led to inactivation of genes which were involved in development. We then further tested the effect of KDM2B on the expression of MOB1. We knocked down KDM2B with shKDM2B-1 and shKDM2B-2 in PANC-1 and AsPC-1. Both shKDM2B-1 and shKDM2B-2 effectively knocked down KDM2B and shKDM2B-2 seemed more efficiency (Fig. 4b and Additional file 10: Figure S6B and S6C). Thus, we chose shKDM2B-2 as shKDM2B in the following studies. The results of western blot and qPCR revealed that knockdown of KDM2B increased the levels of MOB1 in both mRNA and protein levels (Fig. 4b and Additional file 10: Figure S6B, S6C and S6E). KDM2B cooperated with Kras, and PANC-1 and AsPC-1 were both with Kras mutation. We further tested the effect of KDM2B on MOB1 in BxPC-3 cells which harbored wild-type Kras. We found that knockdown of KDM2B also increased the expression of MOB1 in BxPC-3. These results indicated that the function of KDM2B was not totally dependent on Kras. Furthermore, we found that knockdown of KDM2B decreased the levels of H3K27me3 and increased the levels of H3K36me2 and H3K4me3 (Fig. 4b and Additional file 10: Figure S6B, S6C and S6D). Further ChIP assay showed that KDM2B and H3K27me3 directly bound to the TSS region of *MOB1* in PANC-1 and AsPC-1 cells. And knockdown of KDM2B decreased the binding of KDM2B and H3K27me3 on *MOB1* promoter (Fig. 4c). But knockdown of KDM2B had little effect on the recruitment of H3K36me2 and H3K4me3 within the MOB1 locus (Additional file 10: Figure S6F).

We then generated *MOB1* promoter reporter pLuc-MOB1 which contained the surround bases of TSS. Luciferase assays showed that knockdown of KDM2B increased the transcriptional activity of the promoter reporter (Fig. 4d). All these data demonstrated that *MOB1* was a direct downstream target of KDM2B which regulated the expression of *MOB1* at the transcriptional level.

### KDM2B regulated hippo pathway and promoted PDAC proliferation, migration and invasion via MOB1

**Fig. 5:**
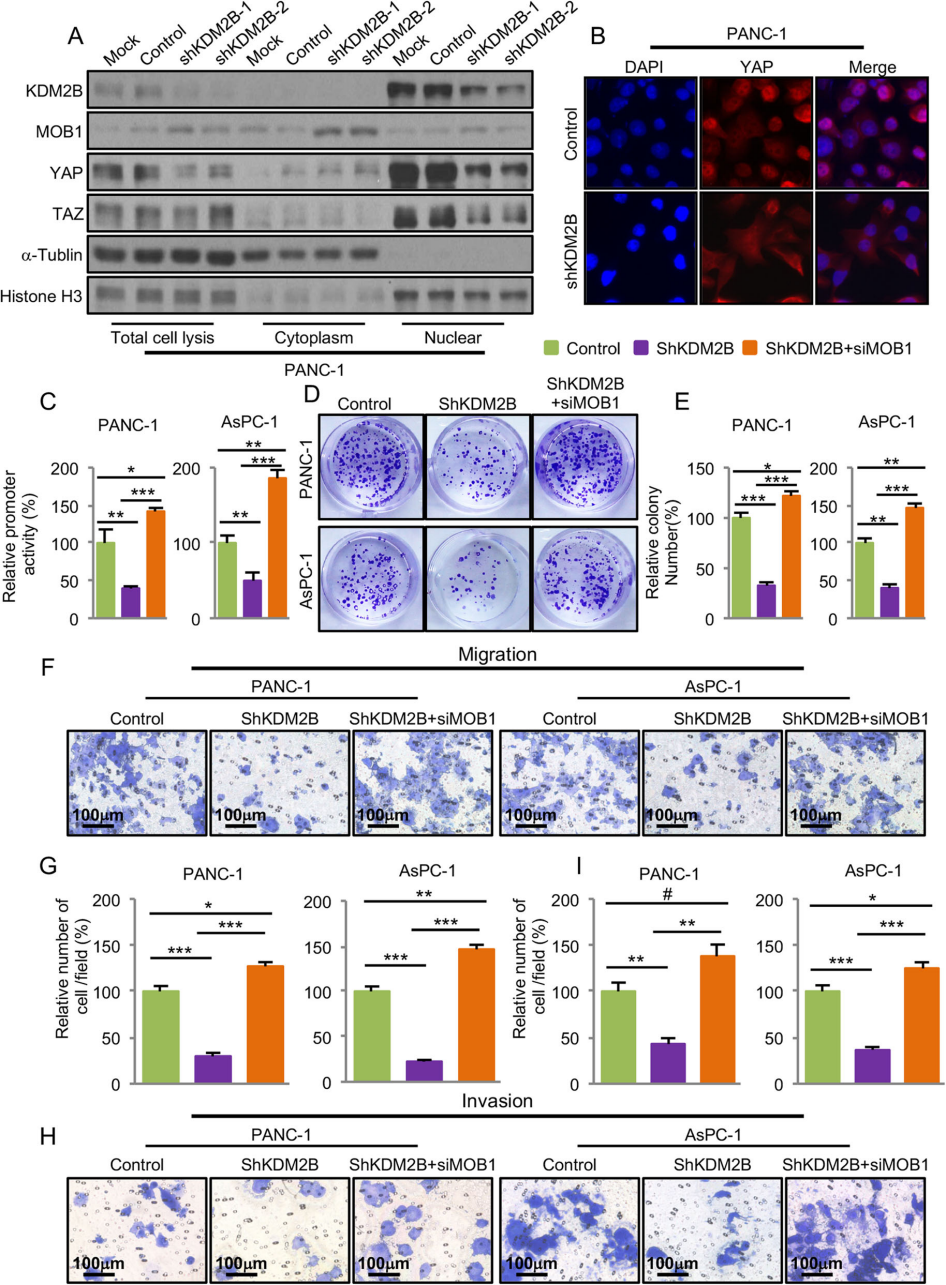
KDM2B regulated Hippo pathway and promoted PDAC proliferation, migration and invasion via MOB1. **a**, PANC-1 cells transfected with KDM2B shRNAs for 48 h. Total cell lysis, cytosolic and nuclear proteins were extracted and the protein levels of KDM2B, MOB1, YAP and TAZ were measured using western blot analysis (α-Tublin was used for internal control of total cell lysis and cytoplasmic fractions, and Histone H1 was used for internal control of nuclear fractions). **b**, PANC-1 cells were transfected with shKDM2B or control vectors for 24 h. Cell immunofluorescence was used to detect YAP (red) in PANC-1 at low cell density (nuclei were stained with DAPI (blue)). **c**, PANC-1 and AsPC-1 cells were co-transfected with 0.2 μg of the 8xGTIIC-Luciferase reporter, control vector or shKDM2B or shKDM2B + siMOB1 for 24 h. Promoter activity was examined using a dual luciferase assay kit. PANC-1 and AsPC-1 cells were transfected with control vectors or shKDM2B or shKDM2B + siMOB1 for 24 h. **d** and **e**, colony formation assay was performed and numbers of colonies were counted 14 days after. **f**, **g**, **h** and **i**, the migration and invasion of PANC-1 and AsPC-1 cells were determined as described in Materials and Methods. Representative tumor cell migrated or invaded were photographed

KDM2B transcriptionally regulated the expression MOB1 and MOB1 was one of the core components of the Hippo pathway. To confirm the regulating roles of KDM2B on the Hippo pathway, we analyzed the impact of KDM2B on the cytoplasm and nuclear protein levels of YAP and TAZ in PANC-1 cells. As we expected, knockdown of KDM2B increased the protein levels of MOB1 in total cell lysis and cytoplasm, and decreased the levels of YAP and TAZ in total cell lysis and nuclear, but increased the levels of YAP and TAZ in cytoplasm (Fig. 5a and Additional file 11: Figure S7A, S7B and S7C). Furthermore, knockdown of KDM2B resulted in cytoplasmic localization of YAP and TAZ at low cell density (Fig. 5b and Additional file 12: Figure S8). We then further tested whether KDM2B regulated YAP and TAZ via MOB1. The result showed that knockdown of KDM2B decreased the protein levels of YAP and TAZ, but knockdown of MOB1 attenuated the effect of shKDM2B on YAP and TAZ and even further increased the levels of YAP and TAZ (Additional file 13: Figure S9A). Further ChIP assay showed that KDM2B and H3K27me3 did not bind to YAP and TAZ promoter regions (Additional file 13: Figure S9B and S9C). These data demonstrated that knockdown of KDM2B increased the expression of MOB1 and inhibited the nuclear translocation of YAP and TAZ.

We then further tested the effects of KDM2B on Hippo pathway transcriptional activity and the expression of downstream target gene. The results showed that knockdown of KDM2B decreased the transcriptional activity of Hippo pathway (Fig. 5c) and the expression of the Hippo pathway typical downstream target connective tissue growth factor (CTGF) (Fig. 4b and Additional file 10: Figure S6B and S6C). Furthermore, knockdown of MOB1 attenuated the effect of shKDM2B on the transcriptional activity of Hippo pathway (Fig. 5c). Biology studies further confirmed that knockdown of KDM2B inhibited PDAC cells proliferation (Fig. 5d and e), migration (Fig. 5f and g) and invasion (Fig. 5h and i), but knockdown of MOB1 attenuated the effect of shKDM2B on PDAC and even further increased PDAC cells proliferation (Fig. 5d and e), migration (Fig. 5f and g) and invasion (Fig. 5h and i). These results demonstrated that KDM2B regulated Hippo pathway via MOB1 and promoted PDAC development and progression.

### Correlation of KDM2B with MOB1 in PDAC tissues

**Fig. 6:**
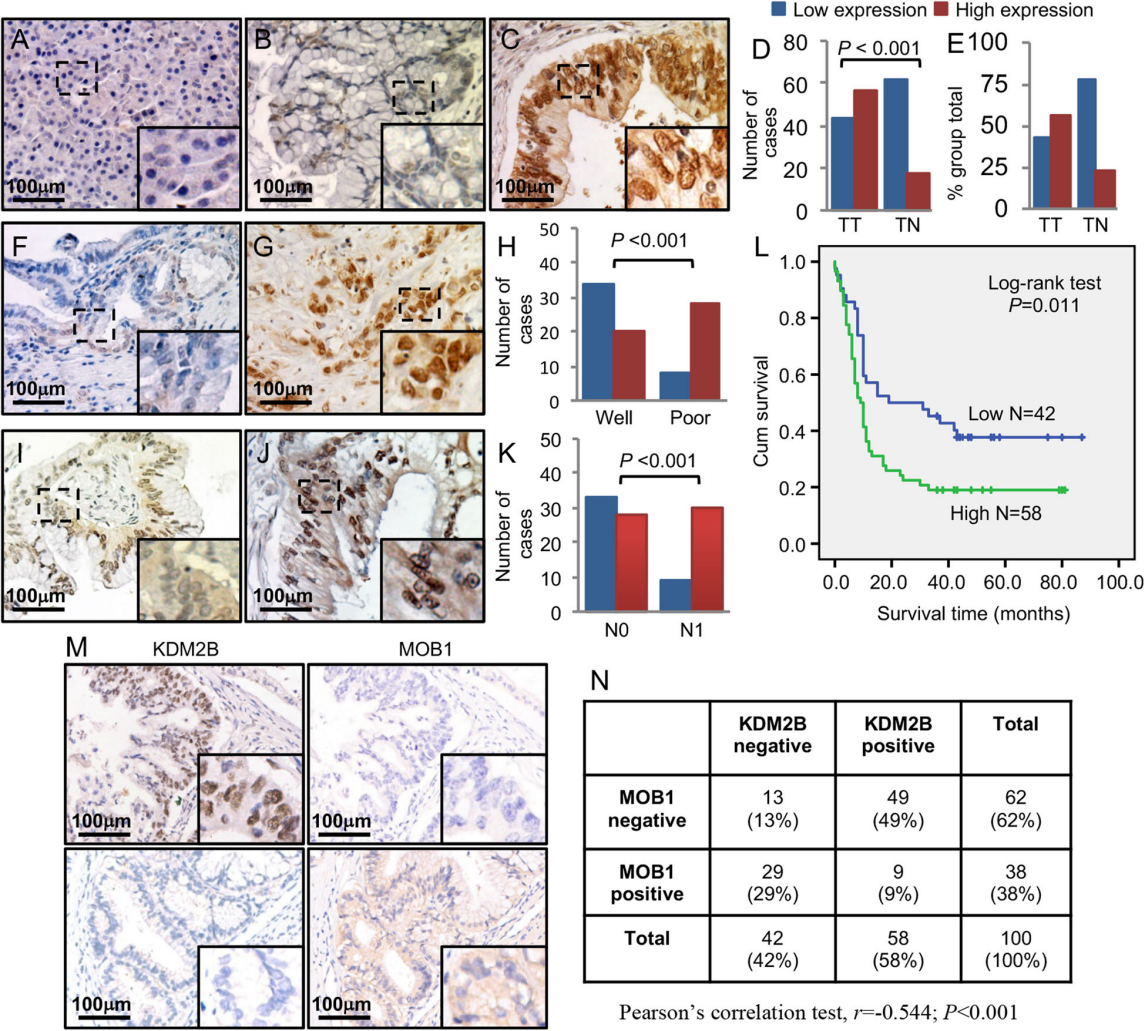
The expressions of KDM2B negative related to MOB1 in PDAC tissues. The same cohorts of TMA sections for MOB1 were immunostained with a specific anti-KDM2B antibody. **a**, representative images of KDM2B low expression in adjacent normal pancreatic tissue specimens. **b**, representative images of KDM2B low expression in PDAC specimens. **c**, representative images of KDM2B high expression in PDAC specimens. **d** and **e**, the expression of KDM2B was significantly higher in tumors (TT) than in adjacent normal tissue (TN). **f** and **g**, representative images of low KDM2B expression in PDAC specimens of well differentiation and high KDM2B expression of poor differentiation. **h**, the expression of KDM2B was positively associated with PDAC poorer differentiation. **i** and **j**, representative images of low KDM2B expression in PDAC specimens without lymph nodes metastasis and high KDM2B expression with lymph nodes metastasis. **k**, the expression of KDM2B positively associated with lymph nodes metastasis. **l**, KDM2B expression was negatively associated with the OS of PDAC patients. **m** and **n**, KDM2B and MOB1 protein expression TMA tissue sections were represented from the cohort. The negative correlation of KDM2B with MOB1 expression was assessed using Pearson correlation coefficient analysis (*n* = 100; *r* = − 0.544, *P* < 0.001)

We have provided evidence that KDM2B transcriptionally suppressed the expression of MOB1. To further confirm our results, we analyzed the protein levels of KDM2B in the serial PDAC tissue array of MOB1 using IHC. We observed KDM2B mainly positive staining in the nucleus of cancer tissues, but negative or weak expression in the adjacent normal tissues (Fig. 6a, b, c, d and e). Furthermore, the increased expression of KDM2B was positively associated with poorer tumor differentiation (Fig. 6f, g, h and Additional file 3: Table S3), lymph nodes metastasis (Fig. 6i, j, k and Additional file 3: Table S3) and higher TNM stages (*P* = 0.001; Additional file 3: Table S3). Kaplan-Meier analysis and log-rank test were used to investigate the prognostic value of KDM2B. In univariate analysis, KDM2B was closely associated with the OS of PDAC patients (*P* = 0.011; Fig. 6l and Additional file 4: Table S4). However, multivariate analysis showed that KDM2B was not an independent prognostic factor of PDAC patients (*P* = 0.065, Additional file 4: Table S4). We then further analyzed the correlated expression of MOB1 and KDM2B. As shown in Fig. 6m and n, direct negative correlation between MOB1 and KDM2B expression was found in PDAC tissues (*r* = − 0.544, *P* < 0.001).

## Discussion

The Hippo pathway is a highly conserved signaling pathway in mammalian. Under physiological conditions, the normal activity of Hippo kinase cascade which mainly contains MST1/2, LATS1/2, MOB1 and SAV1, YAP and TAZ are restricted to a low level of expression and activity [38]. But in cancer cells, they override the negative regulation of MST1/2, LATS1/2, MOB1 and SAV1, and result in inactivation of Hippo kinase cascade which leads to nuclear translocation of YAP and TAZ and the expression of downstream targets [39]. Many studies have revealed that YAP and TAZ were independent prognostic factors for PDAC patients and promoted PDAC initiation and progression [12, 40–44]. It also has been reported that Glucose Sensor O-GlcNAcylation, miR-181c, lncRNA MALAT1 and UCA1 promoted PDAC development, progression and chemoresistance via Hippo signaling [45–48]. The roles of YAP and TAZ have been widely studied, however, the expression, function and regulation of other core components of Hippo signaling, such as MOB1, still need further study.

MOB1 is an adaptor protein, which is in complex with MST1/2 and LATS1/2, and acts as the co-activator of LATS1/2 [38]. In Akira Suzuki group’s study, *MOB1a/1b* double-mutant mice resulted in tumor development and upregulation of MOB1 inhibited several types of cancer development and progression, including osteosarcoma, hepatocellular carcinoma (HCC) and glioblastoma et al. [3, 6, 18]. In PDAC, it has been reported that miR-181c directly repressed MST1, LATS2, SAV1 and MOB1 expression and contributed to PDAC chemoresistance via Hippo signaling [46]. In another study, lncRNA UCA1 interacted with MOB1, LATS1 and YAP and promoted PDAC migration and invasion [48]. Till now, at least six different MOB proteins which are encoded by independent genes are reported in human [5]. In Min Chen group’s study, they found that MST1-MOB1 complex could be disrupted by MST4-MOB4 complex which played a pro-oncogenic role in PDAC [48]. In our study, we found that the expression of MOB1 was decreased in PDAC cell lines and tissues, and was negatively associated with poorer tumor differentiation, lymph nodes metastasis, higher TNM stages and patients’ OS. This was the first time to investigate the expression level of MOB1 in PDAC and revealed that MOB1 was an independent prognostic factor of PDAC patients. We further found that restored expression of MOB1 suppressed the proliferation, migration and invasion of PDAC. These data revealed that MOB1 also acted as a tumor suppressor of PDAC. To further demonstrate the molecular mechanisms of the tumor-suppressive function of MOB1 in PDAC, we focused on the impact of MOB1 on Hippo signaling. We found that MOB1 increased the phosphorylation of YAP and TAZ, inhibited their nuclear translocation and decreased Hippo signaling transcriptional activity. It has been reported that MOB1 is in complex with MST1/2 and LATS1/2, and further promoted the phosphorylation of LATS1/2. In our study, we found that MOB1 bound to MST1/2 and LATS1/2, increased total levels of LATS1 and further increased the phosphorylation of LATS1 when MST1/2 were activated with OA. These data indicated that MOB1 was an adaptor in the complex with MST1/2 and LATS1/2 in PDAC. Furthermore, a negative correlation of MOB1 level and YAP nuclear expression in the same cohort was found in PDAC tissues. These data demonstrated that MOB1 inhibited PDAC progression via activation of Hippo signaling. We then further investigated the mechanism of decreased expression of MOB1 in PDAC.

It has been reported that ubiquitin ligase praja2 led to proteolysis of MOB1 in glioblastoma [18]. In acute lymphoid leukemia cells, CpG DNA methylation in *MOB1* promoters inhibited the expression of *MOB1* gene [37]. In PDAC, miRNA-181c directly bound to the 3’UTR region of *MOB1* transcripts and inhibited the expression level [46]. In our study, we treated PDAC cell lines with demethylating agent 5-aza and found that 5-aza increased the protein levels of MOB1. These data indicated that CpG DNA methylation also suppressed the expression of *MOB1* gene in PDAC. In PDAC, it has been reported that KDM2B interacted with EZH2, bound to the TSSs of a series of genes, decreased H3K27me3 and inactivated the genes which were involved in development [26]. Furthermore, the promoters of these genes were highly methylated [26].Thus, we tested the effect of KDM2B on MOB1 expression. Our data showed that knockdown of KDM2B led to increased expression and promoter transcriptional activity of *MOB1* gene. ChIP assay further confirmed that KDM2B directly bound to the promoter region of MOB1. We further tested the effect of KDM2B on regulating Hippo pathway and PDAC progression via MOB1. The results showed that knockdown of KDM2B inhibited YAP and TAZ nuclear translocation and Hippo pathway transcriptional activity. Furthermore, knockdown of KDM2B suppressed PDAC cells proliferation, migration, and invasion, whereas knockdown of MOB1 attenuated the suppression effect of shKDM2B on Hippo pathway transcriptional activity and PDAC proliferation, migration and invasion. To further confirm our results, we analyzed the expression of KDM2B and MOB1 in PDAC array with the same cohort, and the result showed a negative correlation between KDM2B and MOB1. These data not only revealed a novel and important mechanism that KDM2B transcriptionally decreased the expression of MOB1, but also demonstrated a critical rule of KDM2B in PDAC. It has been reported that deletion of G-protein-coupled receptor-48 (GPR48) led to decreased expression of KDM2B through cAMP-CREB pathway [49]. Furthermore, miR-448 and miR-146a-5p could also regulate the expression of KDM2B [50, 51]. However, the mechanism of increased expression of KDM2B in PDAC has not been revealed, which could be further studied.

## Conclusions

In summary, this study for the first time provided both clinical and mechanistic evidence supporting that suppression of KDM2B on MOB1 promoted PDAC progression via Hippo pathway. Therefore, we have not only identified a new valuable biomarker for PDAC and demonstrated the underling mechanism of decreased expression of MOB1, but also found a promising molecular target for new therapeutic strategies for controlling PDAC progression.

## Supplementary information

**Additional file 1: Table S1.** Correlation between the clinicopathologic characteristics and MOB1 expression (*n* = 100).

**Additional file 2: Table S2.** Correlation between the clinicopathologic characteristics and YAP expression (*n* = 100).

**Additional file 3: Table S3.** Correlation between the clinicopathologic characteristics and KDM2B expression (*n* = 100).

**Additional file 4: Table S4.** Summary of univariate and multivariate Cox regression analysis of overall survival duration in all PADC tissues.

**Additional file 5: Figure S1.** Knocking down efficiency of siRNAs of MOB1. Knockdown of MOB1 with siRNAs, and the mRNA levels of MOB1 were verified by qPCR.

**Additional file 6: Figure S2.** The effect of MOB1 on PDAC migration and invasion.

**Additional file 7: Figure S3.** MOB1 binding to MST1/2 and LATS1/2 in PDAC.

**Additional file 8: Figure S4.** Relative expression levels of Fig. 3. A and B, relative expression levels of Fig. 3a, c and d, relative expression levels of Fig. 3b

**Additional file 9: Figure S5.** Expression of YAP in and its association with clinicopathologic features of PDAC.

**Additional file 10: Figure S6.** KDM2B transcriptionally suppressed the expression of MOB1. A, relative expression levels of Fig. 4a, b and c, relative expression levels of Fig. 4b

**Additional file 11: Figure S7.** Relative expression levels of Fig. 5a, b and c, relative expression levels of Fig. 5.

**Additional file 12: Figure S8.** KDM2B regulated TAZ nuclear translocation.

**Additional file 13: Figure S9.** KDM2B regulated YAP and TAZ through MOB1.

## Data Availability

All data generated or analysed during this study are included in this published article.

## Abbreviations

ChIP: Chromatin immunoprecipitation
Co-IP: Co-Immunoprecipitation.
DMEM: Dulbecco’s modified Eagle’s medium
EZH2: Enhancer of zeste homolog 2
FBS: Fetal bovine serum
HDACs: Histone deacetylases
HDMs: Histone demethylases
HMTs: Histone methyltransferases
KDM2B: Lysine Demethylase 2B
LATS1/2: Large tumor suppressor 1/2
MOB1: Mps1 binding protein
PBS: Phosphate-buffered saline
PCR: Polymerase chain reaction
PDAC: Pancreatic ductal adenocarcinoma
shRNA: short hairpin RNA
TAZ: PDZ-binding motif
TMA: Tissue microarray
YAP: Yes Associated Protein
