# Supplementary material for: Lysine demethylase 2 (KDM2B) regulates hippo pathway via MOB1 to promote pancreatic ductal adenocarcinoma (PDAC) progression
Source: J Exp Clin Cancer Res. 2020 Jan 15;39:13. doi: 10.1186/s13046-019-1489-0 (PMC6961382; doi:10.1186/s13046-019-1489-0)
Supplement: Supplementary file 1 — Additional file 1: Table S1. Correlation between the clinicopathologic characteristics and MOB1 expression (n = 100). [file 13046_2019_1489_MOESM1_ESM.docx]

Table S1

Correlation between the clinicopathologic characteristics and MOB1 expression (*n* = 100)

| Clinicopathological parameters | No.of patients | MOB1 expression (n, %) | | |
| --- | --- | --- | --- | --- |
|  |  | Low | High | *P*-value |
| **Cases** | 100 | 62 (62.0) | 38 (38.0) |  |
| **Age (years)** | | | | |
| ≤60 | 50 | 31 (62.0) | 19 (38.0) | 1.0^a^ |
| >60 | 50 | 31 (62.0) | 19 (38.0) |  |
| **Gender** | | | | |
| Male | 63 | 41 (65.1) | 22 (34.9) | 0.522^a^ |
| Female | 37 | 21 (56.8) | 16 (43.2) |  |
| **Tumor location** | | | | |
| Head | 70 | 39 (55.7) | 31 (44.3) | 0.071^a^ |
| Body and tail | 30 | 23 (76.7) | 7 (23.3) |  |
| **Tumor size (cm)** | | | | |
| ≤3 | 31 | 17 (54.8) | 14 (45.2) | 0.376^a^ |
| >3 | 69 | 45 (65.2) | 24 (34.8) |  |
| **Tumor differentiation** | | | | |
| Well and moderate | 64 | 30 (46.9) | 34 (53.1) | <0.001^*b^ |
| Poor | 36 | 32 (88.9) | 4 (11.1) |  |
| **Nerve invasion** | | | | |
| Negative | 40 | 26 (65.0) | 14 (35.0) | 0.677^a^ |
| Positive | 60 | 36 (60.0) | 24 (40.0) |  |
| **Invasion depth** | | | | |
| T1+T2 | 79 | 50 (63.3) | 29 (36.7) | 0.621^a^ |
| T3+T4 | 21 | 12 (57.1) | 9 (42.9) |  |
| **Lymph nodes metastasis** | | | | |
| N0 (negative) | 61 | 29 (47.5) | 32 (52.5) | <0.001 ^*a^ |
| N1 (positive) | 39 | 33 (84.6) | 6 (15.4) |  |
| **Distant metastasis** | | | | |
| Absent | 98 | 60 (61.2) | 38 (38.8) | 0.524^b^ |
| Present | 2 | 2 (100.0) | 0 (0.0) |  |
| **Clinical stages** | | | | |
| Early stages (≤IIa) | 59 | 27 (45.8) | 32 (54.2) | <0.001 ^*a^ |
| Advanced stages (>IIa) | 41 | 35 (85.4) | 6 (14.6) |  |

^a^Chi-square test; ^b^Fisher's exact test; ^*^*P* < 0.05 indicates a significant association among the variables.
