# Supplementary material for: Lysine demethylase 2 (KDM2B) regulates hippo pathway via MOB1 to promote pancreatic ductal adenocarcinoma (PDAC) progression
Source: J Exp Clin Cancer Res. 2020 Jan 15;39:13. doi: 10.1186/s13046-019-1489-0 (PMC6961382; doi:10.1186/s13046-019-1489-0)
Supplement: Supplementary file 2 — Additional file 2: Table S2. Correlation between the clinicopathologic characteristics and YAP expression (n = 100). [file 13046_2019_1489_MOESM2_ESM.docx]

Table S2

Correlation between the clinicopathologic characteristics and YAP expression (*n* = 100)

| Clinicopathological parameters | No.of patients | YAP expression (n, %) | | |
| --- | --- | --- | --- | --- |
|  |  | Low | High | *P*-value |
| **Cases** | 100 | 43 (43.0) | 57 (57.0) |  |
| **Age (years)** | | | | |
| ≤60 | 50 | 20 (40.0) | 30 (60.0) | 0.686^a^ |
| >60 | 50 | 23 (46.0) | 27 (54.0) |  |
| **Gender** | | | | |
| Male | 63 | 26 (41.3) | 37 (58.7) | 0.680^a^ |
| Female | 37 | 17 (45.9) | 20 (54.1) |  |
| **Tumor location** | | | | |
| Head | 70 | 31 (44.3) | 39(55.7) | 0.826^a^ |
| Body and tail | 30 | 12 (40.0) | 18(60.0) |  |
| **Tumor size (cm)** | | | | |
| ≤3 | 31 | 10 (32.3) | 21 (67.7) | 0.191^a^ |
| >3 | 69 | 33 (47.8) | 36 (52.2) |  |
| **Tumor differentiation** | | | | |
| Well and moderate | 64 | 36 (56.2) | 28 (43.8) | 0.001^*a^ |
| Poor | 36 | 7 (19.4) | 29 (80.6) |  |
| **Nerve invasion** | | | | |
| Negative | 40 | 19 (47.5) | 21 (52.5) | 0.538^a^ |
| Positive | 60 | 24 (40.0) | 36 (60.0) |  |
| **Invasion depth** | | | | |
| T1+T2 | 79 | 34 (43.0) | 45 (57.0) | 1.0^a^ |
| T3+T4 | 21 | 9 (42.9) | 12 (57.1) |  |
| **Lymph nodes metastasis** | | | | |
| N0 (negative) | 61 | 31 (50.8) | 30 (49.2) | 0.063 ^a^ |
| N1 (positive) | 39 | 12 (30.8) | 27 (69.2) |  |
| **Distant metastasis** | | | | |
| Absent | 98 | 42 (42.9) | 56 (57.1) | 1.0^b^ |
| Present | 2 | 1 (50.0) | 1 (50.0) |  |
| **Clinical stages** | | | | |
| Early stages (≤IIa) | 59 | 30 (50.8) | 29 (49.2) | 0.067 ^a^ |
| Advanced stages (>IIa) | 41 | 13 (31.7) | 28 (68.3) |  |

^a^Chi-square test; ^b^Fisher's exact test; ^*^*P* < 0.05 indicates a significant association among the variables.
