# Supplementary material for: Lysine demethylase 2 (KDM2B) regulates hippo pathway via MOB1 to promote pancreatic ductal adenocarcinoma (PDAC) progression
Source: J Exp Clin Cancer Res. 2020 Jan 15;39:13. doi: 10.1186/s13046-019-1489-0 (PMC6961382; doi:10.1186/s13046-019-1489-0)
Supplement: Supplementary file 3 — Additional file 3: Table S3. Correlation between the clinicopathologic characteristics and KDM2B expression (n = 100). [file 13046_2019_1489_MOESM3_ESM.docx]

Table S3

Correlation between the clinicopathologic characteristics and KDM2B expression (*n* = 100)

| Clinicopathological parameters | No.of patients | KDM2B expression (n, %) | | |
| --- | --- | --- | --- | --- |
|  |  | Low | High | *P*-value |
| **Cases** | 100 | 42 (42.0) | 58 (58.0) |  |
| **Age (years)** | | | | |
| ≤60 | 50 | 25 (50.0) | 25 (50.0) | 0.156^a^ |
| >60 | 50 | 17 (34.0) | 33 (66.0) |  |
| **Gender** | | | | |
| Male | 63 | 27 (42.9) | 36 (57.1) | 0.837^a^ |
| Female | 37 | 15 (40.5) | 22 (59.5) |  |
| **Tumor location** | | | | |
| Head | 70 | 31 (44.3) | 39(55.7) | 0.515^a^ |
| Body and tail | 30 | 11 (36.7) | 19(63.3) |  |
| **Tumor size (cm)** | | | | |
| ≤3 | 31 | 18 (58.1) | 13 (41.9) | 0.048^*a^ |
| >3 | 69 | 24 (34.8) | 45 (65.2) |  |
| **Tumor differentiation** | | | | |
| Well and moderate | 64 | 34 (53.1) | 30 (46.9) | 0.003^*a^ |
| Poor | 36 | 8 (22.2) | 28 (77.8) |  |
| **Nerve invasion** | | | | |
| Negative | 40 | 17 (42.5) | 23 (57.5) | 1.0^a^ |
| Positive | 60 | 25 (41.7) | 35 (58.3) |  |
| **Invasion depth** | | | | |
| T1+T2 | 79 | 31 (39.2) | 48 (60.8) | 0.325^a^ |
| T3+T4 | 21 | 11 (52.4) | 10 (47.6) |  |
| **Lymph nodes metastasis** | | | | |
| N0 (negative) | 61 | 33 (54.1) | 28 (45.9) | 0.002 ^*a^ |
| N1 (positive) | 39 | 9 (23.1) | 30 (76.9) |  |
| **Distant metastasis** | | | | |
| Absent | 98 | 42 (42.9) | 56 (57.1) | 0.508^b^ |
| Present | 2 | 0 (0.0) | 2 (100.0) |  |
| **Clinical stages** | | | | |
| Early stages (≤IIa) | 59 | 33 (55.9) | 26 (44.1) | 0.001 ^*a^ |
| Advanced stages (>IIa) | 41 | 9 (22.0) | 32 (78.0) |  |

^a^Chi-square test; ^b^Fisher's exact test; ^*^*P* < 0.05 indicates a significant association among the variables.
