# Supplementary material for: Lysine demethylase 2 (KDM2B) regulates hippo pathway via MOB1 to promote pancreatic ductal adenocarcinoma (PDAC) progression
Source: J Exp Clin Cancer Res. 2020 Jan 15;39:13. doi: 10.1186/s13046-019-1489-0 (PMC6961382; doi:10.1186/s13046-019-1489-0)
Supplement: Supplementary file 4 — Additional file 4: Table S4. Summary of univariate and multivariate Cox regression analysis of overall survival duration in all PADC tissues. [file 13046_2019_1489_MOESM4_ESM.docx]

Supplementary Table S4

Summary of univariate and multivariate Cox regression analysis of overall survival duration in all PADC tissues

| Clinicopathological parameters | Cox-regression analysis | | | |
| --- | --- | --- | --- | --- |
|  | Univariate analysis | | Multivariate analysis | |
|  | HR 95% CI | *P*-value | HR 95% CI | *P*-value |
| **MOB1 expression** | | | | |
| Low | 1.000 | <0.001^*^ | 1.000 | <0.001^*^ |
| High | 4.501（2.568-7.886） |  | 4.134(2.297-7.442) |  |
| **YAP expression** | | | | |
| Low | 1.000 | <0.001^*^ | 1.000 | 0.001^*^ |
| High | 0.393（0.239-0.645） |  | 0.418(0.248-0.705) |  |
| **KDM2B expression** | | | | |
| Low | 1.000 | 0.011^*^ | 1.000 | 0.065 |
| High | 0.539（0.333-0.875） |  | 0.629(0.385-1.029) |  |
| **Age (years)** | | | | |
| ≤60 | 1.000 | 0.274 |  |  |
| >60 | 0.774（0.489-1.226） |  |  |  |
| **Gender** | | | | |
| Male | 1.000 | 0.444 |  |  |
| Female | 1.208（0.744-1.960） |  |  |  |
| **Tumor location** | | | | |
| Head | 1.000 | 0.828 |  |  |
| Body and tail | 0.947（0.578-1.551） |  |  |  |
| **Tumor size (cm)** | | | | |
| ≤3 | 1.000 | 0.850 |  |  |
| >3 | 1.048（0.643-1.710） |  |  |  |
| **Tumor differentiation** | | | | |
| Well and moderate | 1.000 | 0.038^*^ | 1.000 | 0.230 |
| Poor | 0.610（0.381-0.978） |  | 0.735(0.445-1.214) |  |
| **Nerve invasion** | | | | |
| Negative | 1.000 | 0.495 |  |  |
| Positive | 0.851（0.535-1.353） |  |  |  |
| **Invasion depth** | | | | |
| T1+T2 | 1.000 | 0.896 |  |  |
| T3+T4 | 1.039（0.588-1.834） |  |  |  |
| **Lymph nodes metastasis** | | | | |
| N0 (negative) | 1.000 | 0.002^*^ | 1.000 | 0.027^*^ |
| N1 (positive) | 0.492（0.309-0.785） |  | 0.571(0.348-0.938) |  |
| **Distant metastasis** | | | | |
| Absent | 1.000 | 0.427 |  |  |
| Present | 0.569（0.139-2.329） |  |  |  |
| **Clinical stages** | | | | |
| Early stages (≤IIa) | 1.000 | 0.001^*^ | 1.000 | 0.010^*^ |
| Advanced stages (>IIa) | 0.473（0.297-0.755） |  | 0.537(0.334-0.862) |  |

HR: hazard ratio; 95% CI: 95% confidence interval; ^*^*P* < 0.05 indicates a significant association among the variables.
