# Supplementary figures and images for: Lysine demethylase 2 (KDM2B) regulates hippo pathway via MOB1 to promote pancreatic ductal adenocarcinoma (PDAC) progression
Source: J Exp Clin Cancer Res. 2020 Jan 15;39:13. doi: 10.1186/s13046-019-1489-0 (PMC6961382; doi:10.1186/s13046-019-1489-0)

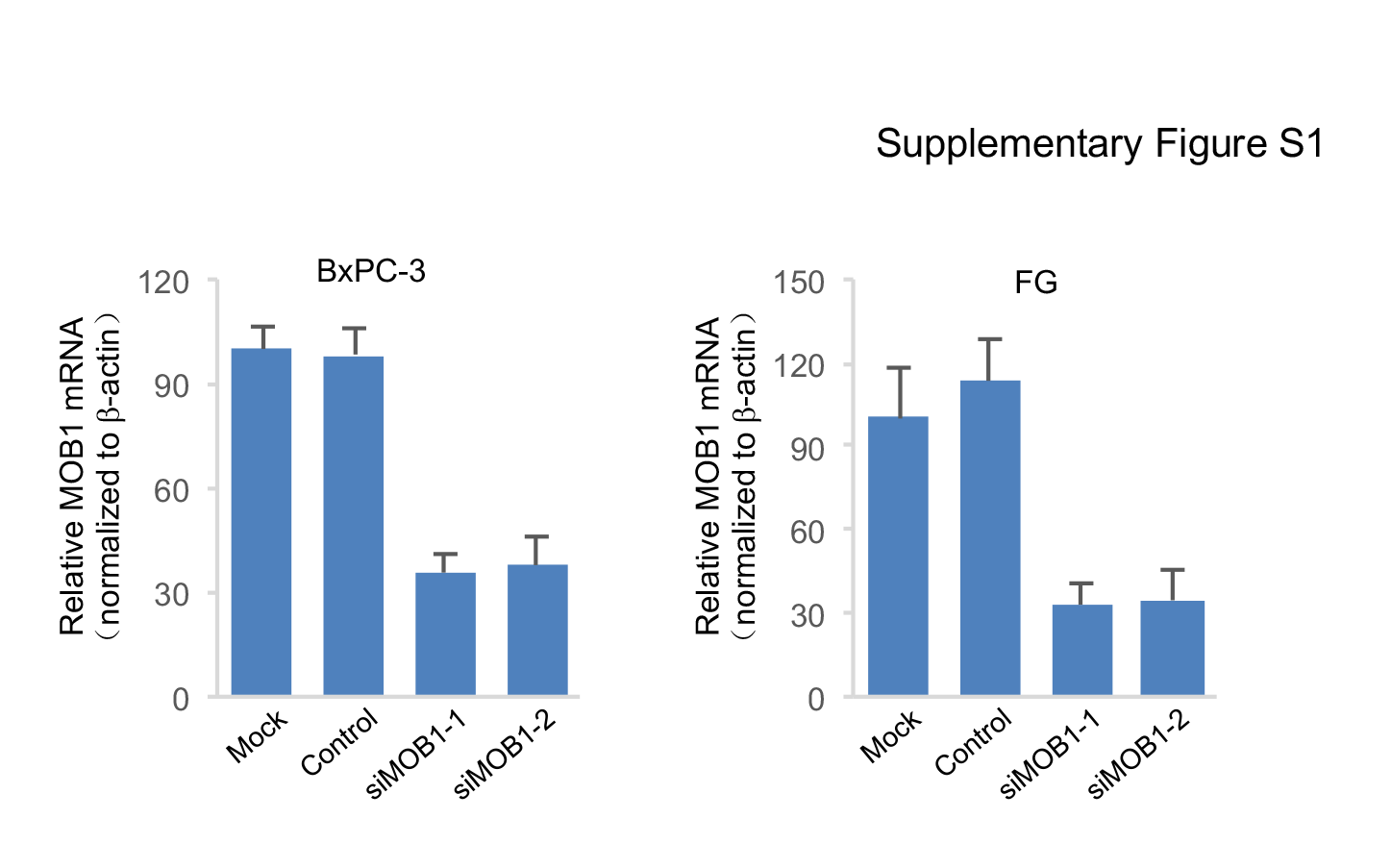

Supplement: Supplementary file 5 — Additional file 5: Figure S1. Knocking down efficiency of siRNAs of MOB1. Knockdown of MOB1 with siRNAs, and the mRNA levels of MOB1 were verified by qPCR. [file 13046_2019_1489_MOESM5_ESM.tif]

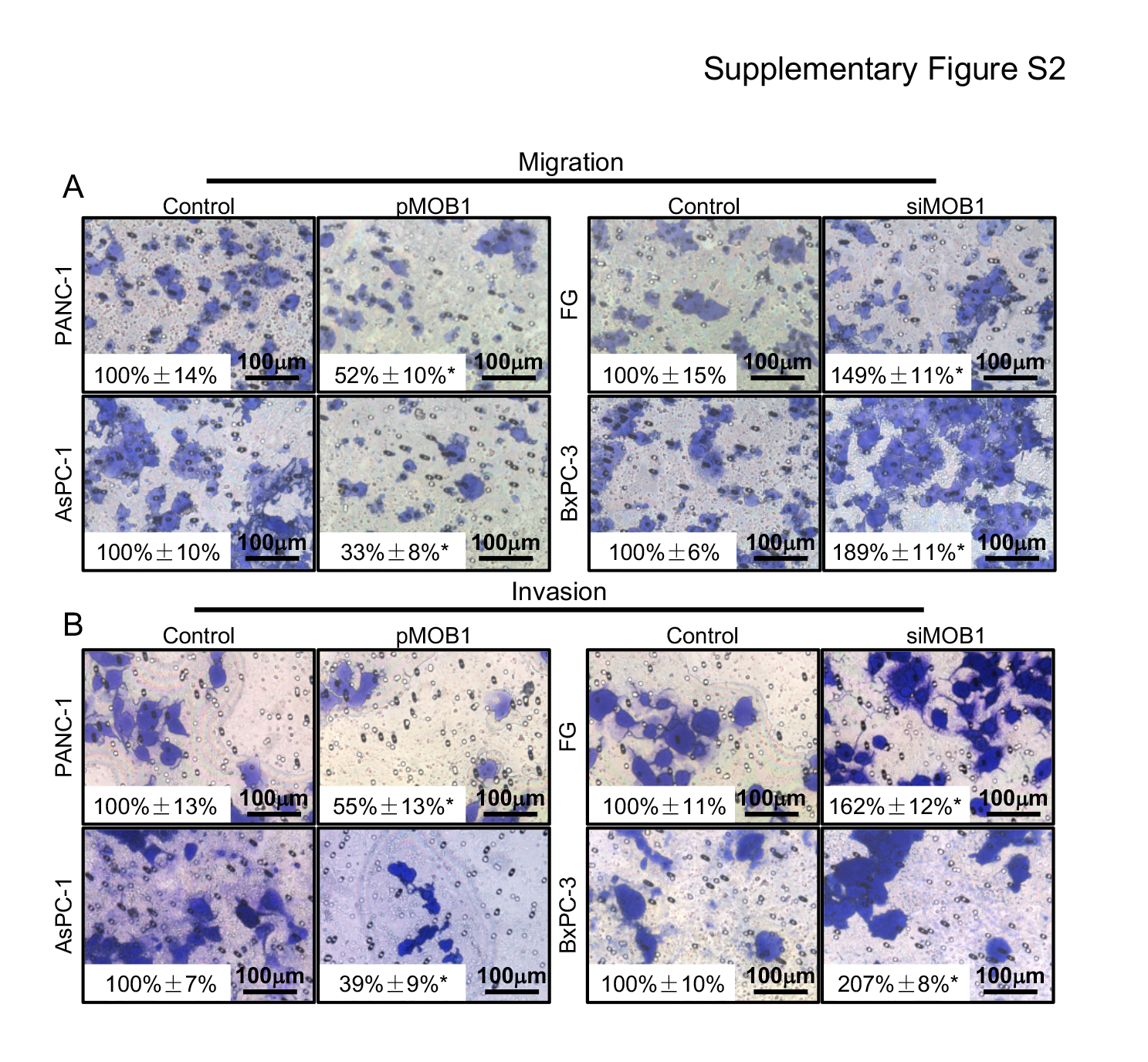

Supplement: Supplementary file 6 — Additional file 6: Figure S2. The effect of MOB1 on PDAC migration and invasion. [file 13046_2019_1489_MOESM6_ESM.tif]

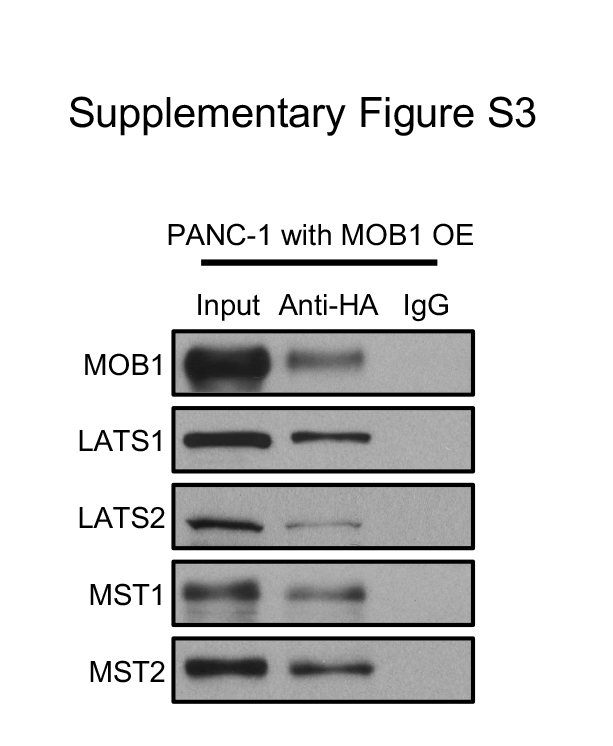

Supplement: Supplementary file 7 — Additional file 7: Figure S3. MOB1 binding to MST1/2 and LATS1/2 in PDAC. [file 13046_2019_1489_MOESM7_ESM.tif]

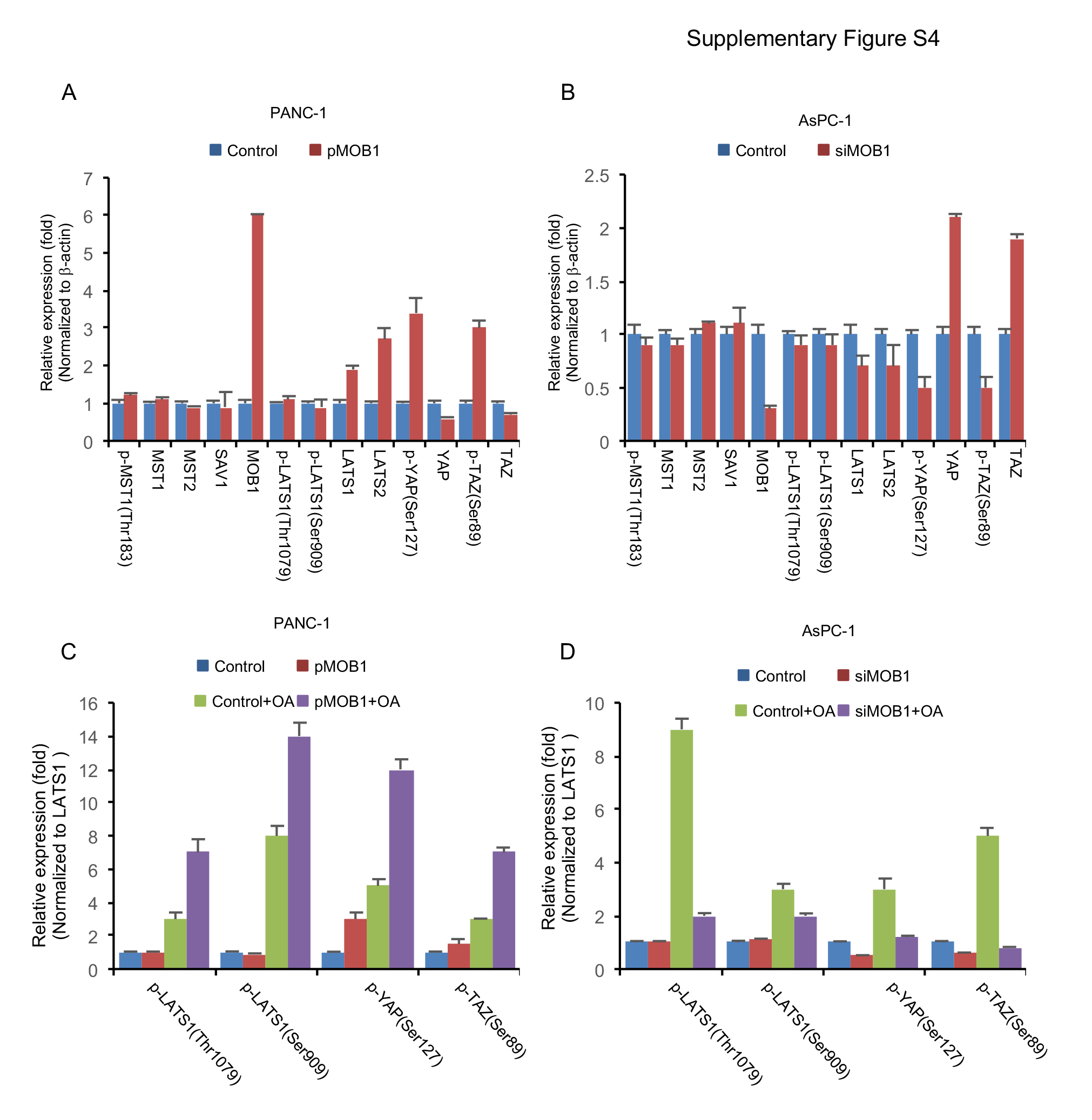

Supplement: Supplementary file 8 — Additional file 8: Figure S4. Relative expression levels of Fig. 3. A and B, relative expression levels of Fig. 3a, c and d, relative expression levels of Fig. 3b [file 13046_2019_1489_MOESM8_ESM.tif]

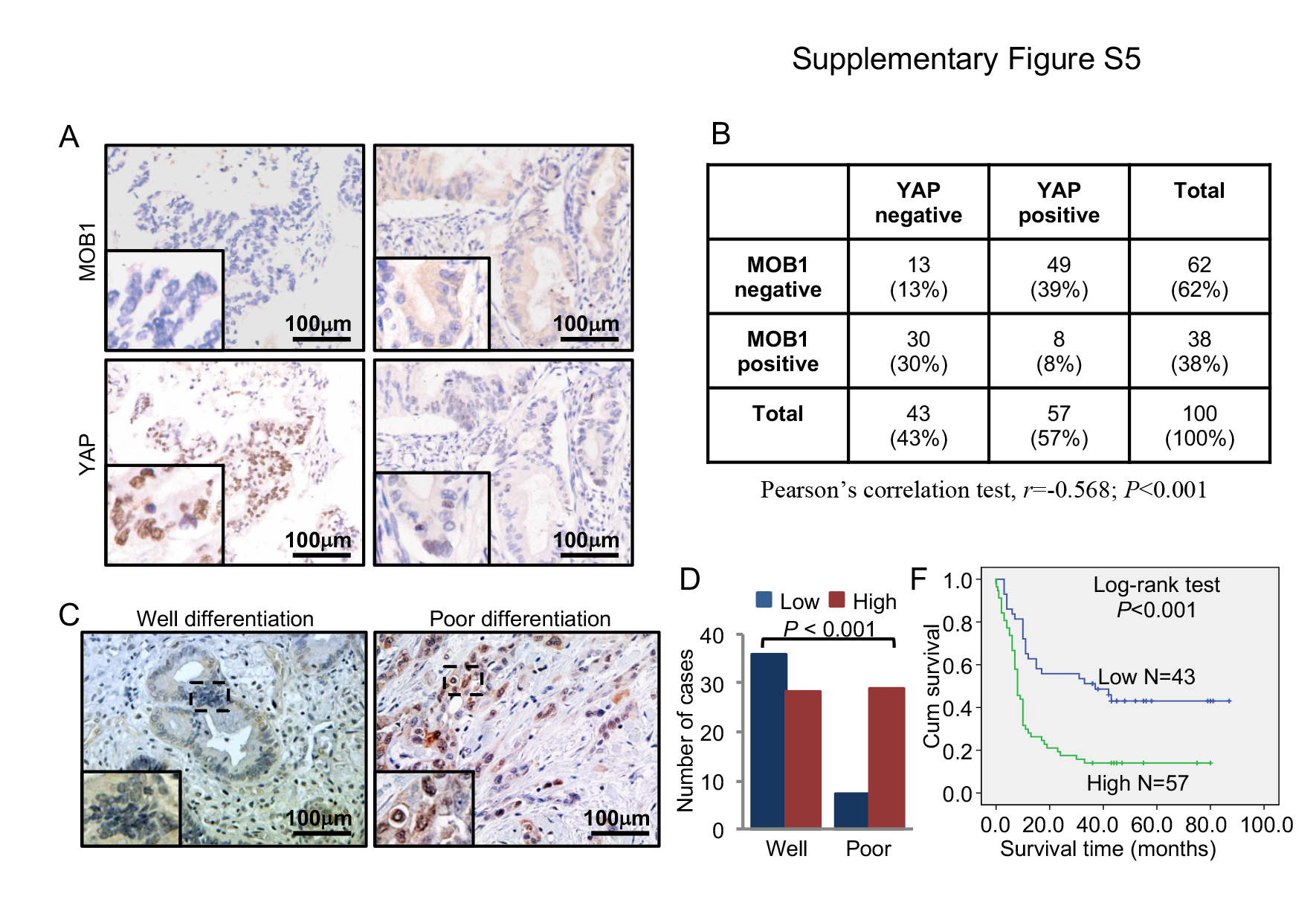

Supplement: Supplementary file 9 — Additional file 9: Figure S5. Expression of YAP in and its association with clinicopathologic features of PDAC. [file 13046_2019_1489_MOESM9_ESM.tif]

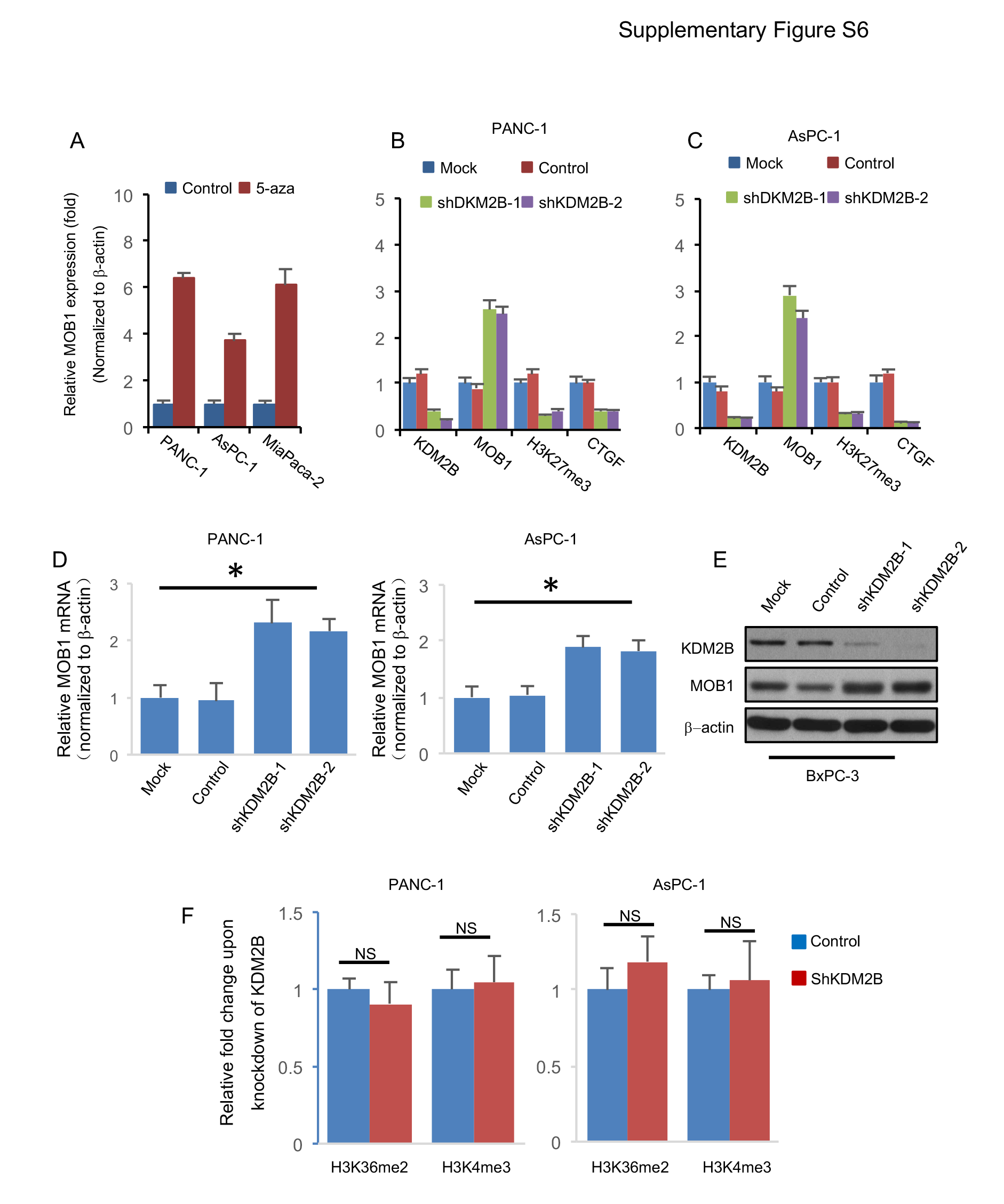

Supplement: Supplementary file 10 — Additional file 10: Figure S6. KDM2B transcriptionally suppressed the expression of MOB1. A, relative expression levels of Fig. 4a, b and c, relative expression levels of Fig. 4b [file 13046_2019_1489_MOESM10_ESM.tif]

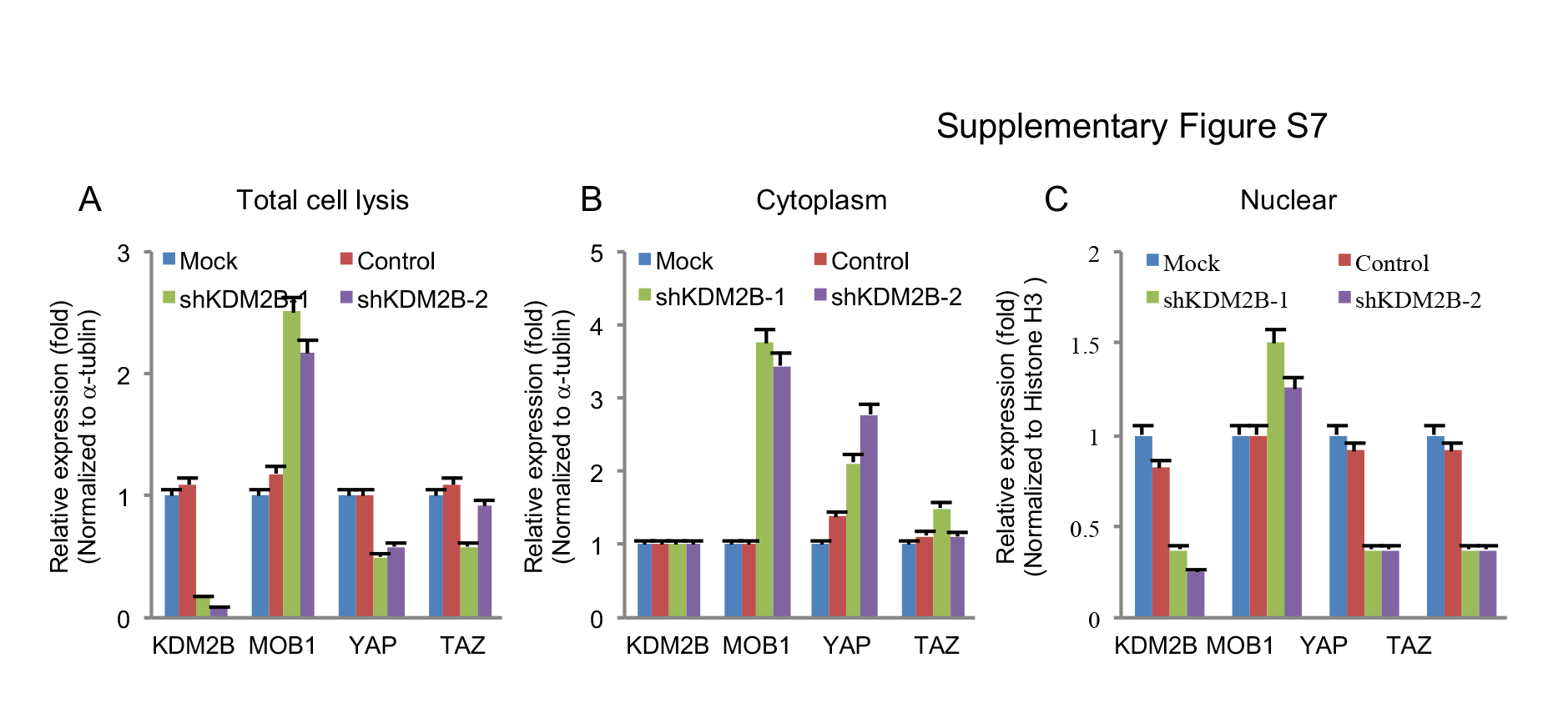

Supplement: Supplementary file 11 — Additional file 11: Figure S7. Relative expression levels of Fig. 5a, b and c, relative expression levels of Fig. 5. [file 13046_2019_1489_MOESM11_ESM.tif]

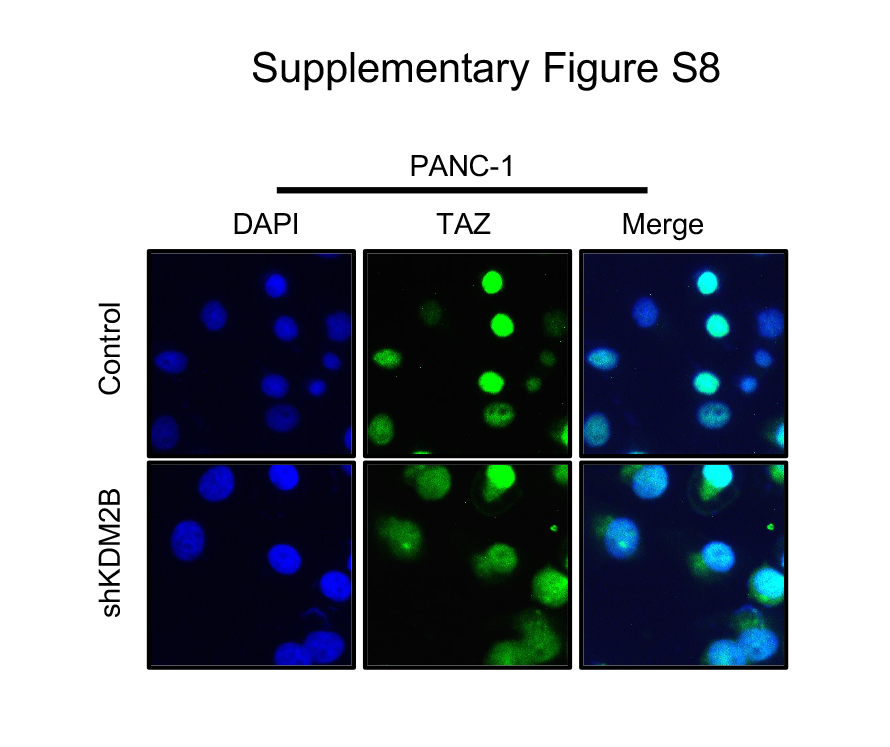

Supplement: Supplementary file 12 — Additional file 12: Figure S8. KDM2B regulated TAZ nuclear translocation. [file 13046_2019_1489_MOESM12_ESM.tif]

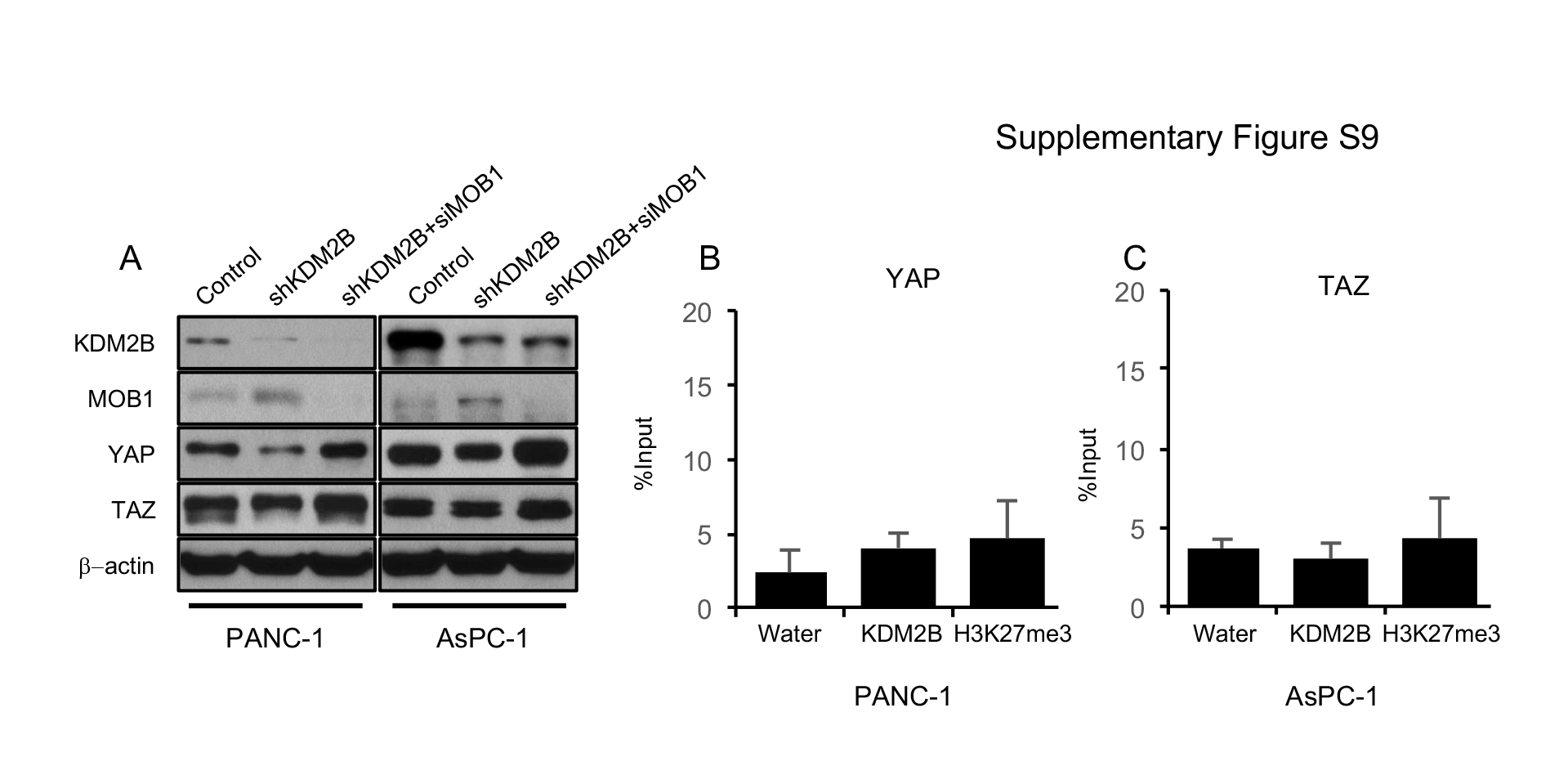

Supplement: Supplementary file 13 — Additional file 13: Figure S9. KDM2B regulated YAP and TAZ through MOB1. [file 13046_2019_1489_MOESM13_ESM.tif]
